# Dependency on the TYK2/STAT1/MCL1 axis in anaplastic large cell lymphoma

**DOI:** 10.1038/s41375-018-0239-1

**Published:** 2018-08-21

**Authors:** Nicole Prutsch, Elisabeth Gurnhofer, Tobias Suske, Huan Chang Liang, Michaela Schlederer, Simone Roos, Lawren C. Wu, Ingrid Simonitsch-Klupp, Andrea Alvarez-Hernandez, Christoph Kornauth, Dario A. Leone, Jasmin Svinka, Robert Eferl, Tanja Limberger, Astrid Aufinger, Nitesh Shirsath, Peter Wolf, Thomas Hielscher, Christina Sternberg, Fritz Aberger, Johannes Schmoellerl, Dagmar Stoiber, Birgit Strobl, Ulrich Jäger, Philipp B. Staber, Florian Grebien, Richard Moriggl, Mathias Müller, Giorgio G. Inghirami, Takaomi Sanda, A. Thomas Look, Suzanne D. Turner, Lukas Kenner, Olaf Merkel

**Affiliations:** 1 Clinical Institute of Pathology, Department for Experimental and Laboratory Animal Pathology, Medical University of Vienna, Vienna, Austria; 2 Department of Pediatric Oncology, Dana-Farber Cancer Institute, Harvard Medical School, Boston, USA; 3 Unit of Laboratory Animal Pathology, University of Veterinary Medicine Vienna, Vienna, Austria; 4 Department of Oncology, Amgen Discovery Research, South San Francisco, CA 94080 USA; 5 Clinical Institute of Pathology, Medical University of Vienna, Vienna, Austria; 6 Institute of Cancer Research, Medical University of Vienna & Comprehensive Cancer Center (CCC), Vienna, Austria; 7 Department of Dermatology and Venereology, Medical University of Graz, Graz, Austria; 8 Division of Biostatistics, German Cancer Research Center (DKFZ), Heidelberg, Germany; 9 Department of Molecular Biology, Cancer Cluster Salzburg, Faculty of Natural Sciences, Paris Lodron University, Salzburg, Austria; 10 Department of Biochemistry, Christian-Albrechts-University Kiel, Kiel, Germany; 11 Ludwig Boltzmann Institute for Cancer Research (LBI-CR), Vienna, Austria; 12 Institute of Pharmacology, Center for Physiology and Pharmacology, Medical University of Vienna, Vienna, Austria; 13 Institute of Animal Breeding and Genetics, University of Veterinary Medicine Vienna, Vienna, Austria; 14 Department of Medicine I, Clinical Division of Hematology and Hemostaseology and Comprehensive Cancer Center (CCC), Medical University of Vienna, Vienna, Austria; 15 Medical University of Vienna, Vienna, Austria; 16 Pathology and Laboratory Medicine, Weill Cornell Medical College, New York, NYC USA; 17 European Research Initiative for ALK related malignancies (www.erialcl.net), Vienna, Austria; 18 Cancer Science Institute of Singapore, National University of Singapore, 117599 Singapore, Singapore; 19 Division of Cellular and Molecular Pathology, Department of Pathology, University of Cambridge, Cambridge, UK; 20 CBMed Core Lab2, Medical University of Vienna, Vienna, Austria

**Keywords:** Targeted therapies, T-cell lymphoma

## Abstract

TYK2 is a member of the JAK family of tyrosine kinases that is involved in chromosomal translocation-induced fusion proteins found in anaplastic large cell lymphomas (ALCL) that lack rearrangements activating the anaplastic lymphoma kinase (ALK). Here we demonstrate that TYK2 is highly expressed in all cases of human ALCL, and that in a mouse model of NPM-ALK-induced lymphoma, genetic disruption of *Tyk2* delays the onset of tumors and prolongs survival of the mice. Lymphomas in this model lacking *Tyk2* have reduced STAT1 and STAT3 phosphorylation and reduced expression of *Mcl1*, a pro-survival member of the BCL2 family. These findings in mice are mirrored in human ALCL cell lines, in which TYK2 is activated by autocrine production of IL-10 and IL-22 and by interaction with specific receptors expressed by the cells. Activated TYK2 leads to STAT1 and STAT3 phosphorylation, activated expression of MCL1 and aberrant ALCL cell survival. Moreover, TYK2 inhibitors are able to induce apoptosis in ALCL cells, regardless of the presence or absence of an ALK-fusion. Thus, TYK2 is a dependency that is required for ALCL cell survival through activation of MCL1 expression. TYK2 represents an attractive drug target due to its essential enzymatic domain, and TYK2-specific inhibitors show promise as novel targeted inhibitors for ALCL.

## Introduction

TYK2 was the first Janus kinase described, and it was shown to collaborate with JAK1 to facilitate interferon-α/β (IFN) responsiveness [1, 2]. Recently, activation of TYK2 has been noted in a number of malignancies including T-cell acute lymphoblastic leukemia (T-ALL), anaplastic large cell lymphoma (ALCL) and nerve sheath tumor [3–6]. In T-ALL cell lines, activating somatic mutations have been detected in the TYK2 FERM domain (G36D, S47N) and in the kinase domain (E957D, R1072H) [3]. Unmutated TYK2 also represented a dependency in T-ALL cell lines and patient samples [3]. Moreover, germline TYK2 mutations potentially causing ALL have been described [7]. Recently, somatic TYK2 fusion proteins have also been detected in ALL [8], AML [9], cutaneous [5], and systemic ALCLs that lack anaplastic lymphoma kinase (ALK) fusion genes [6]. Despite involvement of TYK2 in fusion proteins and the presence of activating mutations in some cancers, with the exception of T-ALL [3, 10], little is known regarding TYK2’s oncogenic functions and downstream effectors. To elucidate the role of TYK2 in tumorigenesis, we focused on ALCL as a well-defined lymphoma subtype [11]. ALCL is a CD30 positive, aggressive non-Hodgkin T-cell lymphoma with early onset that is characterized in approximately half of all patients (ALCL, ALK+) by fusion of the catalytic domain of *ALK* with the N-terminus of the gene encoding the Nucleophosmin 1 (NPM1) protein due to a *t*(2;5) chromosomal translocation [11]. Despite initial classification as a T-cell lymphoma arising in mature memory T cells, several recent publications point toward a transformation of early thymic progenitor cells in ALCL [12, 13]. ALCL, ALK+ patients can be effectively treated with the poly-chemo- therapy (e.g., CHOP) or ALK inhibitors. However, still 25–30% of patients relapse leading to very aggressive disease [14, 15]. An additional targeted agent is provided by the recently introduced armed CD30 antibody brentuximab vedotin, which shows good responses but is often associated with polyneuropathy as a severe side effect [16]. ALCL patients without ALK translocations cannot be treated by ALK inhibitors and have a worse prognosis compared to ALCL, ALK+ patients creating an urgent need for new and refined molecularly targeted therapeutic options for ALCL [15, 17, 18]. The WHO has classified ALCL, ALK− as a distinct disease with sub-entities defined by chromosomal rearrangements that disrupt the DUSP22 and TP63 tumor suppressors [18]. Several transplant but also transgenic mouse models for ALCL, ALK+ have been created, with the *CD4*-NPM-ALK transgenic mouse being the best established [19–21]. Similar to ALK, TYK2 is a tyrosine kinase that can be readily inhibited by small molecules and therefore represents an attractive therapeutic target in ALCL.

We show here that the TYK2 tyrosine kinase is expressed in human ALCLs irrespective of ALK status and is essential for tumor cell viability. Genetic studies in a transgenic NPM-ALK driven lymphoma model also demonstrate that T cell-specific loss of Tyk2 delays the onset of tumors and prolongs the survival of mice. We furthermore show that TYK2 is activated by an autocrine loop involving IL-10 and IL-22 and that STAT1 and STAT3 are essential mediators of aberrant tumor cell survival through activation of the pro-survival protein MCL1. Our data underscore the potential therapeutic importance in ALCL of TYK2 inhibitors which are currently in late preclinical stages of development.

## Materials and methods

### Cell culture

ALCL cell lines were obtained from DSMZ, Braunschweig, Germany. For cytokine complementation experiments, recombinant human Interleukin-10 (10 ng/ml, rh IL-10, Immunotools, Friesoythe, Germany) or rhIL-22 (20 ng/ml, Immunotools) were used. For detection of downstream targets, ALCL cells were incubated with TYK2 inhibitors or pan-JAK inhibitors (including 1 µM JAK inhibitor I, Calbiochem, San Diego, CA, USA) for 3 or 6 h and then incubated with IFN-α for 10 min before immunoblot analysis. Description of quantitative RT-PCR, flow cytometry, cytokine arrays and immunohistochemistry, shRNA sources, *CRISPR*/*Cas9* genome editing and murine lymphoma models can be found in Supplementary Methods.

### Cloning of mutant TYK2 construct and rescue experiment

Retroviral constructs encoding the mutant TYK2_E957D cDNA as well as the WT TYK2 cDNA were obtained from Dr. Takaomi Sanda from CSI, Singapore. Production of retrovirus expressing TYK2_E957D and TYK2_WT was performed as previously described [3].

### Cell growth and viability assays

For cell counting of shRNA knockdown or CRISPR knockout experiments, cells were seeded into 12-well plates in triplicates at day 1 and counted at days 2, 3, 4, and 5. For drug treatment, cells were incubated with TYK2 inhibitors or pan-JAK inhibitors (Table S6) for 72 h, and cell proliferation was quantified using the XTT Cell Proliferation Assay Kit (ATCC, Manassas, Virginia, USA) according to manufacturer’s instructions. All values were normalized to the untreated control.

### Immunoblotting

Cells were lysed in RIPA buffer containing phosphatase and protease inhibitors. Equivalent amounts of protein were diluted in sample buffer and separated by 10% SDS-PAGE. Proteins were transferred to nitrocellulose membranes (Millipore), subjected to immunoblot analysis and stained with antibodies as listed in Table S4. Western blot quantification was conducted using Image J version 2007.

## Results

### TYK2 ablation in *CD4-*NPM-ALK transgenic mice reduces the growth rate of lymphoma and significantly increases survival

**Fig. 1:**
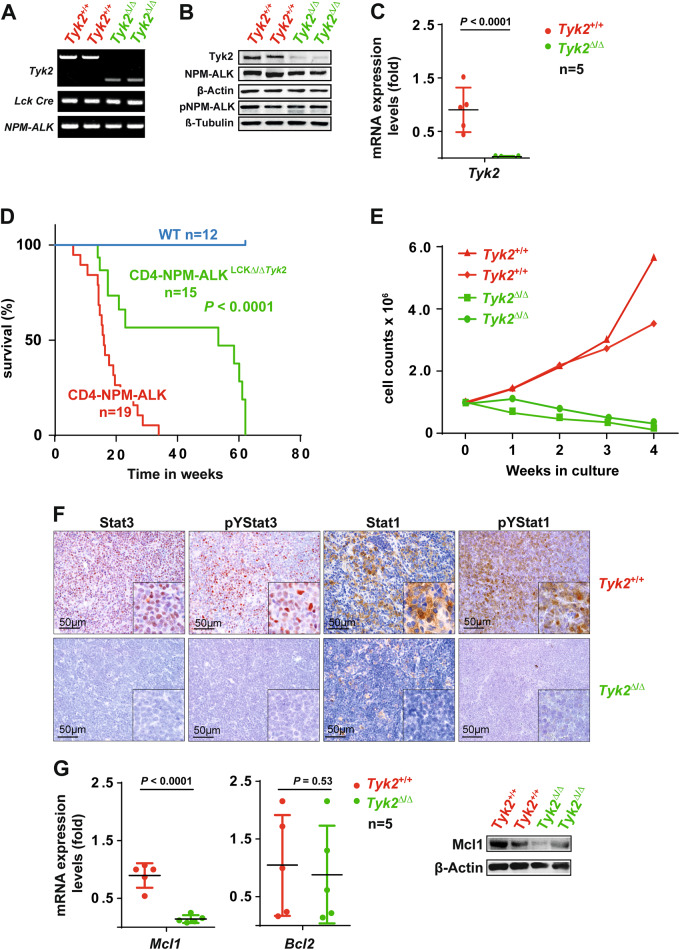
Conditional TYK2 knockout prolongs survival of *CD4*-NPM-ALK^LCKΔΔTyk2^ transgenic mice. **a** *Tyk2* alleles assessed by PCR in DNA isolated from murine *CD4*-NPM-ALK or *CD4*-NPM-ALK^LCKΔΔTyk2^ lymphomas. **b** Western blot analysis of Tyk2, NPM-ALK, p-NPM-ALK, and β-Actin/β-Tubulin expression in mouse lymphomas. **c** *Tyk2* mRNA expression levels in *CD4*-NPM-ALK or *CD4*-NPM-ALK^LCKΔΔTyk2^ lymphomas. Data are mean values ± s.d. of five mice. **d** Kaplan–Meier survival analysis of *CD4*-NPM-ALK and *CD4*-NPM-ALK^LCKΔΔTyk2^ mice. **e** In vitro growth rates of *CD4*-NPM-ALK or *CD4*-NPM-ALK^LCKΔΔTyk2^ lymphoma cells, showing *Tyk2* dependency. Two cell lines per genotype are shown. **f** IHC of lymphoma tissues showing pYStat3 and pYStat1 expression in *CD4*-NPM-ALK mice and the lack of expression in *CD4*-NPM-ALK^LCKΔΔTyk2^ mice. **g** mRNA expression of *Mcl1* and *Bcl2* in *CD4*-NPM-ALK lymphomas and the lack of *Mcl1* expression in *CD4*-NPM-ALK^LCKΔΔTYK2^ lymphomas. Data are mean values ± s.d. of five mice. Western blot shows Mcl1 expression in murine *CD4*-NPM-ALK lymphomas and lack of Mcl1 expression in *CD4*-NPM-ALK^LCKΔΔTyk2^ lymphomas. Compare with *Tyk2* expression depicted in **b**

Chromosomal translocations produce fusion proteins that activate the TYK2 tyrosine kinase in human ALCL that lack activation of ALK [3, 5, 6, 8, 9]. This led us to test the hypothesis that TYK2 plays a unique role in ALCL and function as a dependency even in cases harboring the NPM-ALK fusion genes. Thus, floxed Tyk2 mice were crossed to mice bearing the CRE-recombinase under the *Lck*-promoter, resulting in mice with T-cell-specific Tyk2 deletion [22]. These mice were then crossed to an ALCL mouse model that expresses human NPM-ALK from the CD4 promoter [21]. Deletion of Tyk2 was confirmed by PCR of the Tyk2 gene locus, western blot of tumor tissue and real-time RT-PCR (Fig. 1a–c). NPM-ALK and pNPM-ALK expression levels were not affected (Fig. 1b). *CD4-*NPM-ALK mice with intact Tyk2 developed aggressive T-cell lymphomas from about 12 weeks *post*-*partum*. Log-rank analysis of Kaplan–Meier survival curves indicated significantly longer survival of *CD4-*NPM-ALK^LCKΔΔTyk2^ as compared to *CD4-*NPM-ALK mice (median survival of 53.3 weeks *CD4-*NPM-ALK^LCKΔΔTyk2^ versus 16.0 weeks in control *CD4-*NPM-ALK mice, *P* < 0.0001, Fig. 1d). To test the ability of tumor cells to grow in an in vitro setting, lymphoma cells from *CD4-*NPM-ALK and *CD4-*NPM-ALK^LCKΔΔTyk2^ mice were taken into culture. *CD4-*NPM-ALK^LCKΔΔTyk2^ lymphomas failed to grow in vitro in contrast to tumor cells isolated from *CD4-*NPM-ALK mice (Fig. 1e). Tumors arising in *CD4-*NPM-ALK^LCKΔΔTyk2^ mice showed increased numbers of apoptotic cells (*P* = 0.034, Suppl. Figure 1A, B), but cell proliferation as assessed by Ki67 staining was not affected (Suppl. Figure 1A, B), suggesting a primary role for Tyk2 in promoting the survival of lymphoma cells. To assess signaling downstream of Tyk2, we examined Stat1 and Stat3 expression at the mRNA and protein levels and found significant reductions of Stat3 and pYStat3 in *CD4*-NPM-ALK^LCKΔΔTyk2^ mice compared to *CD4*-NPM-ALK mice. Stat1 and pYStat1 levels were also decreased in Tyk2 knockout lymphomas at both the mRNA and protein levels (Fig. 1f, Suppl. Figure 1C, D). To elucidate the mechanism of cell survival mediated by Tyk2, expression levels of Bcl2 family proteins were assessed, in particular Mcl1, which is pivotal for ALCL cell survival [23]. Interestingly, in lymphomas from *CD4*-NPM-ALK^LCKΔΔTyk2^ mice, MCL1 expression was decreased at both the mRNA and protein levels as compared to *CD4*-NPM-ALK mice expressing TYK2 (*P* < 0.0001; Fig. 1g). Loss of Tyk2 did not affect Bcl2 expression levels in these lymphoma cells.

### Inhibition of TYK2 by gene knockdown induces death of human ALCL cells

**Fig. 2:**
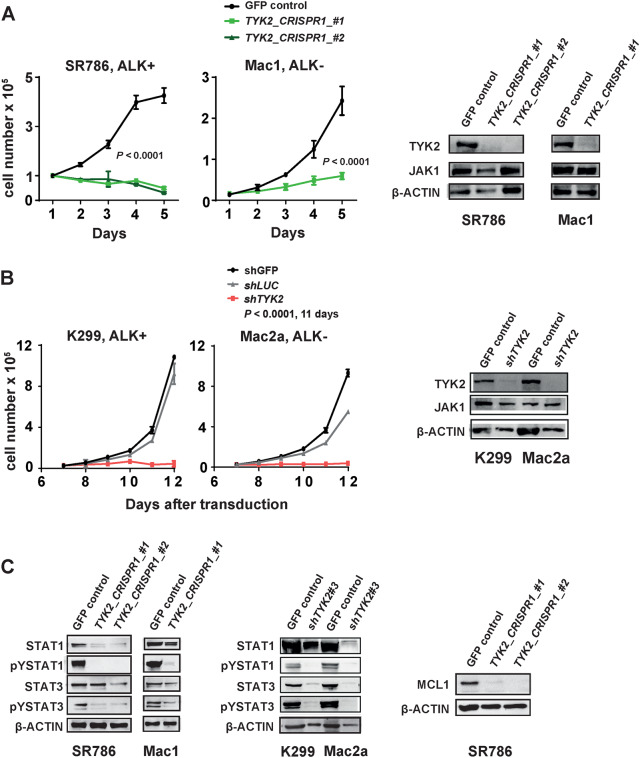
ALCL cells depend on TYK2 for survival. **a** CRISPR knockout of TYK2 using sgRNA targeting of the FERM Domain (TYK2_CRISPR1) decreases cell viability in human ALCL cell lines. Means and standard errors of three experiments are shown (*P* < 0.0001). Western blot analysis of TYK2, JAK1, and beta-ACTIN in the indicated clones verifies specific knockout. **b** Knockdown of TYK2 by lentivirus-transduced shRNAs decreases cell viability in ALCL cell lines. Data show the means and standard errors of three experiments. Expression of TYK2, JAK1, and β-ACTIN was assessed by western blot. **c** Expression of STAT1, pYSTAT1, STAT3, pYSTAT3, and β-ACTIN (control) in SR786 and Mac1 human ALCL cell lines 14 days after sgRNA targeting of *TYK2* or *GFP* (control). Compare with TYK2 expression depicted in **a**. Whole-cell extracts of ALCL cell lines were collected 8 days after shRNA transduction and subjected to immunoblot analysis with the indicated antibodies. Compare with TYK2 expression depicted in the right panel of **b**. Right panel of **c** shows western blot analysis of MCL1 and β-ACTIN in the SR786 cell line 7 days after sgRNA targeting of TYK2 or GFP (control). Compare with TYK2 expression depicted in **a**

To determine whether human ALCL cells depend on TYK2 for survival, we depleted TYK2 using both CRISPR-cas9 and shRNA techniques. The TYK2 protein is comprising of four functional domains, the FERM (F for 4.1 protein, Ezrin, Radixin and Moesin), SH2 (Src Homology 2), pseudo-kinase and kinase domain (Suppl. Figure 2A). Employing CRISPR/Cas9 technology, a disruption was generated in the coding sequences of the FERM domain (TYK2-CRISPR1) or in the kinase domain (TYK2- CRISPR2) of the TYK2 gene. After TYK2-CRISPR1 disruption, single clones lacking TYK2 expression were validated by immunoblot analysis. Remarkably, loss of TYK2 through a *STOP codon* in the FERM domain (TYK2-CRISPR1) resulted in severe growth retardation indicative of TYK2 dependency in two different ALCL cell lines representing NPM-ALK-positive (SR786) and ALK-negative (Mac1, ALK−) lines (Fig. 2a). When Mac1, ALK− cells were injected subcutaneously into NSG mice, TYK2-positive cell clones developed tumors within 2 weeks while TYK2-negative cell clones did not (Suppl. Figure 2B). We employed a different strategy for the TYK2-CRISPR_#2 (kinase domain) knockout in which the ALCL cell lines stably expressed CAS9 and we monitored for successful TYK2-CRISPR2 transduction via a reporter vector expressing GFP-tagged CRISPR2 over three to 5 weeks compared to the same reporter vector containing a GFP-tagged non-targeting guide RNA (NTC) as a control. In all five cell lines tested expression of GFP was lost over time in the TYK2-CRISPR2 guide but not in NTC-control transduced cells, indicating reduced growth due to inactivation of TYK2 in the TYK2-CRISPR2 containing cells (Suppl. Figure 2C). Cell dependency on TYK2 was conclusively shown by rescue of cell survival after co-transduction of a mutated form of TYK2 (E957D, Suppl. Figure 2D) that was not recognized by CRISPR2. In a complementary approach, shRNAs targeting the kinase domain of TYK2-mediated downregulation of TYK2, as confirmed by western blot, and each led to growth reduction and apoptosis induction in both ALCL cell lines tested (Fig. 2b, Suppl. Figure 2E, F). Survival of the cells was rescued by co-expression of the hyperactive form of TYK2 (E957D) that lacked sequences recognized by shRNA TYK2#3 (Suppl. Figure 2G).

Janus kinases such as TYK2 phosphorylate STAT proteins on the critical tyrosine residue, which in turn mediate signal transduction and efficient transcription in the nucleus [24]. Hence, to assess the effect of TYK2 depletion on STAT expression levels and activity, immunoblot analysis of ALCL cell lines was performed with TYK2 depleted by either CRISPR/Cas9 or TYK2-specific shRNA (Fig. 2c). In each of the four human ALCL cell lines, a reduction in levels of phosphorylated STAT1 and STAT3 were observed (Fig. 2c), consistent with the role of heterodimeric JAK proteins containing TYK2 in the phosphorylation of STAT1 and STAT3 in ALCL. To follow-up on the effects of TYK2 on MCL1 expression levels in transgenic mice, we assessed MCL1 expression levels in SR786 ALK+ ALCL cells after TYK2 depletion. As in the mouse model, we found that expression levels of MCL1 were profoundly downregulated by TYK2 depletion, suggesting that TYK2’s ability to promote lymphoma cell survival may be mediated through MCL1 (Fig. 2c).

### Small molecule inhibition of TYK2 induces cell death of ALCL cells

**Fig. 3:**
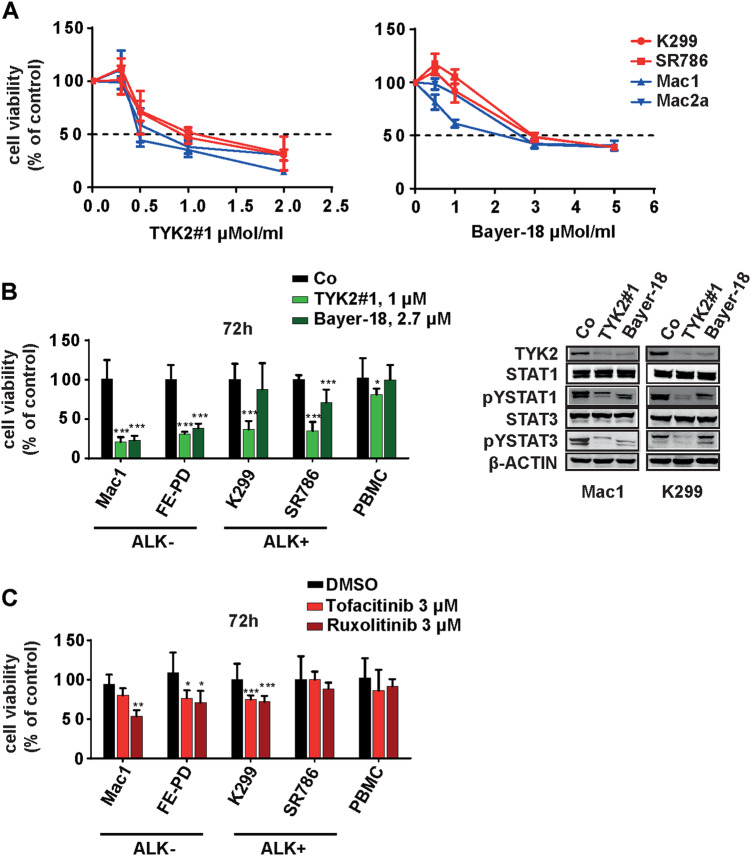
TYK2 and pan-JAK inhibitors reduce viability and pYSTAT1/3 expression in human ALCL cells. **a** The indicated human ALCL cell lines were cultured with graded concentrations of TYK2 inhibitors (TYK2#1 or Bayer-18) for 3 days. Cell viability values are means ± SEM given as a percentage of the untreated control. Values represent the mean of three experiments. **b** The indicated cell lines were treated with the TYK2 inhibitors TYK2#1 (1 μM) or Bayer-18 (2.7 μM), or **c** the pan-JAK inhibitors Ruxolitinib (3 μM) or Tofacitinib (3 μM) for 72 h and cell proliferation was assessed by an XTT assay. Western blot analysis of the indicated antibodies after 48 h of inhibitor treatment

Because ALCL cells are dependent on TYK2 for cell survival, we tested the activity of the recently published small molecule TYK2 inhibitors TYK2#1 [25] and Bayer-18 (Symansis). Initially, we established the 72-h IC_50_ values of the TYK2 inhibitors for four different ALCL cell lines. We found that TYK2#1 and Bayer-18 had IC_50_ values ranging from 0.5–1 µM to 2–3 µM for the different cell lines (Fig. 3a). Then we assayed each of the inhibitors at their mean IC_50_ concentrations in ALCL cell lines against freshly isolated PBMCs. Treatment with TYK2#1 for 72 h reduced cell viability by a mean of 73.4 ± 2.0% in the ALCL, ALK- cell lines Mac1, Mac2a, and FE-PD and 64.5 ± 2.7% in the ALCL, ALK+ cell lines Karpas-299, SR786, and SUDHL-1, whereas PBMCs from healthy donors were only slightly or not affected (Fig. 3b, Suppl. Figure 3A). Bayer-18 also had minimal activity against PBMCs and had comparable activity to TYK2#1 in ALK- ALCL but was less active in ALK+ ALCL cell lines (ALK- 70.1 ± 2.1% versus ALK+ 20.6 ± 5.6% reduction in viability; Fig. 3b, Suppl. Figure 3A). This discrepancy between TYK2#1 and Bayer-18 may be explained by the only partial inhibition of pSTAT1 and pSTAT3 by Bayer-18 in the ALCL ALK+ cell line (Fig. 3b). By contrast, the pan-JAK inhibitors Tofacitinib and Ruxolitinib were less active in ALCL (Tofacitinib 22.0 ± 2.4% and Ruxolitinib 28.8 ± 4.1% viability reduction, Fig. 3c). Neither TYK2#1 nor Bayer-18 had any effect on NPM-ALK or pNPM-ALK expression levels (Suppl. Figure 3B).

We assayed for the levels of apoptosis by Annexin V staining after 48 h of exposure to the drugs, and documented apoptosis induction in ALCL cell lines Mac1, K299, and SR786 (TYK2#1 66.1 ± 5.9% and Bayer-18 50.8 ± 10.0% Annexin V staining; Suppl. Figure 3B). We assayed the downstream consequences of TYK2 inhibition by assessing the levels using western blotting of TYK2, STAT1, STAT3, pYSTAT1, and pYSTAT3. Surprisingly, total TYK2 levels were reduced 48 h after treatment with either inhibitor. Drug-induced decreased expression of total TYK2 may be due to reduced protein stability in the absence of auto-phosphorylation, as previously described [26] (Fig. 3b). Unfortunately, as in T-ALL, endogenous phospho-TYK2 was not detectable in ALCL cells when using currently available reagents that have limited sensitivity [3]. We found downregulation of pYSTAT1 and to a lesser extent of pYSTAT3 after inhibiting TYK2, whereas total protein levels of STAT1 and STAT3 were not affected.

### STAT1 and STAT3 are TYK2 targets promoting tumor growth in ALCL

**Fig. 4:**
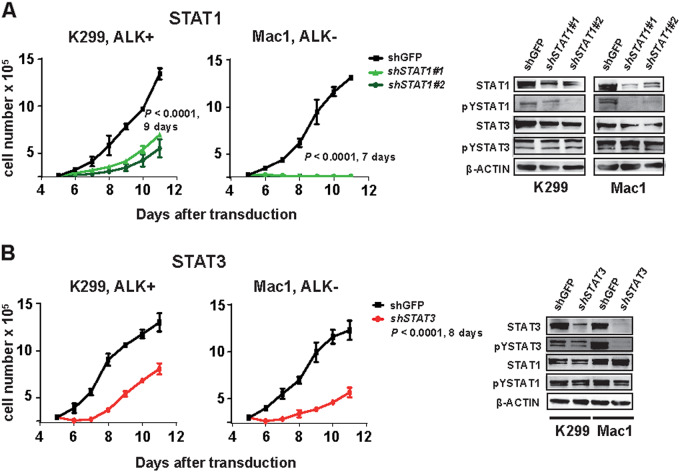
Depletion of STAT1 or STAT3 leads to reduced growth of human ALCL cells. **a** Knockdown of STAT1 by lentivirus-transduced shRNAs decreases cell viability in ALCL cell lines. Means ± SEM of three experiments are shown. Cells with and without STAT1 knockdown were subjected to immunoblot analysis using antibodies for STAT3, pYSTAT3, STAT1, pYSTAT1, and β- ACTIN controls. **b** Knockdown of STAT3 by lentivirus-transduced shRNAs decreases cell viability in ALCL cell lines. Means ± SEM of three experiments are shown. Whole-cell extracts of cells with shRNA-mediated STAT3 knockdown were subjected to immunoblot analysis using antibodies for STAT3, pYSTAT3, STAT1, pYSTAT1, and β-ACTIN controls

Because depletion or small molecule mediated inhibition of TYK2 strongly affected STAT1 signaling, we studied the effects of depletion of STAT1 on cell survival. Transduction with vectors encoding shRNAs targeting STAT1 showed a marked reduction of STAT1 by western blotting and profoundly reduced cell growth rate (Fig. 4a). Then we transduced five ALCL cell lines with a GFP-tagged CRISPR/Cas9 STAT1 deletion construct and showed that GFP-positive cells were depleted over time but not the non-targeting control (NTC) transduced cells, indicating that STAT1 was essential for ALCL cell survival (Suppl. Figure 4A). Indeed, shRNA-mediated depletion of STAT1 had a greater effect on cell death, as determined by positive staining for Annexin V, than depletion of STAT3 (Fig. 4b and Suppl. Figure 4B). To compare the TYK2-specific (Bayer-18 or TYK2) and the pan-JAK inhibitor, JAK inhibitor 1 or Ruxolitinib, we treated ALCL cells for 3 h, and then stimulated the cells for 10 min with Interferon-alpha (IFN-α), which is known to induce TYK2 and JAK1 activity [1, 27]. Treatment with the pan-JAK inhibitor JAK inhibitor 1 led to abrogation of both pYSTAT1 and pYSTAT3 activation but the TYK2-specific inhibitor only affected inhibition of pYSTAT1 and not pYSTAT3 (Suppl. Figure 4C). To clarify the effect of TYK2 loss on the downstream effector STAT1, we performed a rescue experiment by expressing wild-type STAT1 in the K299_TYK2ko cell line. Strikingly, expressing wild-type STAT1 could partially restore the viability of the K299_TYK2ko cell line; whereas, the STAT1 Y701F plasmid, which is incapable of being activated through phosphorylation, did not show any effect (Suppl. Figure 4D). Suppl. Figure 4E shows that TYK2 ko results in decreased STAT1 and pYSTAT1 compared to the CRISPR control cell line, whereas cells rescued with wild-type STAT1 exhibit restored levels of STAT1 and pYSTAT1.

### IL-10 and IL-22 are mediators of TYK2 activity and both are critical for ALCL cell survival

**Fig. 5:**
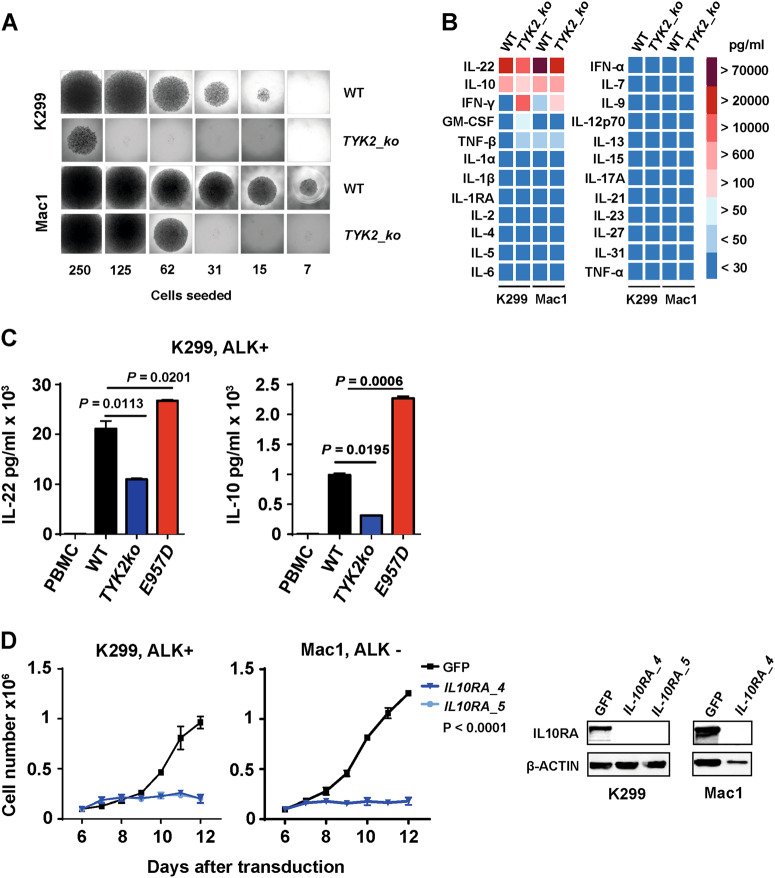
TYK2 activity induces expression of IL-10 and IL-22 in human ALCL. **a** Limiting dilution of the ALK-positive cell line Karpas-299 or the ALK-negative cell line Mac1 with and without CRISPR-Cas9 *TYK2* knockout in 96-well plates containing RPMI1640 and 10% FCS. *TYK2* knockout (TYK2_ko) cells require greater plating cell numbers for cell growth assessed after 2 weeks of incubation. **b** Heat map panel of cytokines detected in the supernatant of ALCL cell lines Karpas-299 and Mac1 with and without TYK2. Cytokine levels were analyzed by ProCarta Multiplexx assay. **c** IL-10 and IL-22 protein expression in the supernatants of the ALK positive cell line Karpas-299 with and without *TYK2* knockout, compared to cells expressing TYK2-E957D and to PBMCs. Supernatants were collected from cell cultures after 48 h and analyzed by the ProCarta Multiplexx assay as above. **d** Knockdown of *IL-10RA* by lentivirus-transduced shRNAs decreases cell viability in ALCL cell lines. Cells were subjected to immunoblot analyses and stained with antibodies against IL10RA and β-ACTIN

JAK-STAT signaling is a key mediator of cytokine production leading to the release of autocrine or paracrine factors that influence differentiation, immune modulation, and survival. Thus, in ALCL signaling mediated through TYK2 may be responsible for the release of autocrine factors that stimulate cell growth. Indeed, TYK2_CRISPR1 knockout cells lacked the ability to grow on limiting dilution (Fig. 5a, Suppl. Figure 5A). These data suggest that the absence of, or a reduction in essential autocrine survival factors produced by the TYK2 knockout cells impacts ALCL cell survival. In order to identify these factors, we performed a comprehensive cytokine screen for 24 selected cytokines that have been previously described in the context of ALCL and lymphoma [3, 28, 29] (Fig. 5b). The most abundant cytokines detected in our screen were IL-22 and IL-10, which are also expressed in primary patient tumors as determined by the analysis of published RNAseq data (Fig. 5b, Suppl. Fig 5A) 6. These two factors are especially intriguing because TYK2 is intimately involved in signal transduction downstream of the IL-22 and IL-10 receptors, acting as a heterodimeric complex with JAK1 [28]. The active role of TYK2 in driving the expression of IL-22 and IL-10 was further confirmed as production of these cytokines was reduced in cells lacking expression of TYK2 (45%, *P* *=* 0.0113 and 63%, *P* *=* 0.0195, respectively), an effect that could be reversed in cells transduced to express the hyperactive E957D mutant of TYK2 (Fig. 5c). Interestingly, both cytokines share a common chain IL-10RB in their heterodimeric receptors: IL-10RA and IL-10RB chains for IL-10 versus IL-22R and IL-10RB for IL-22 [30]. Using shRNA constructs, expression of IL-10RA or IL-10RB were knocked-down in ALCL cell lines showing a reduction in cell growth in both cases (Fig. 5d, Suppl. Figure 5B,C). Taken together, these data point toward an autocrine mechanism mediated by TYK2 in which ALCL cells produce IL-10 and IL-22, which bind to their receptors on the same cells, activating an autocrine TYK2-mediated single transduction pathway resulting in pYSTAT1 with efficient translocation and gene regulation in the nucleus, where it is essential for survival of the cells and autonomous cell growth.

### TYK2 is expressed in ALCL regardless of ALK status

**Fig. 6:**
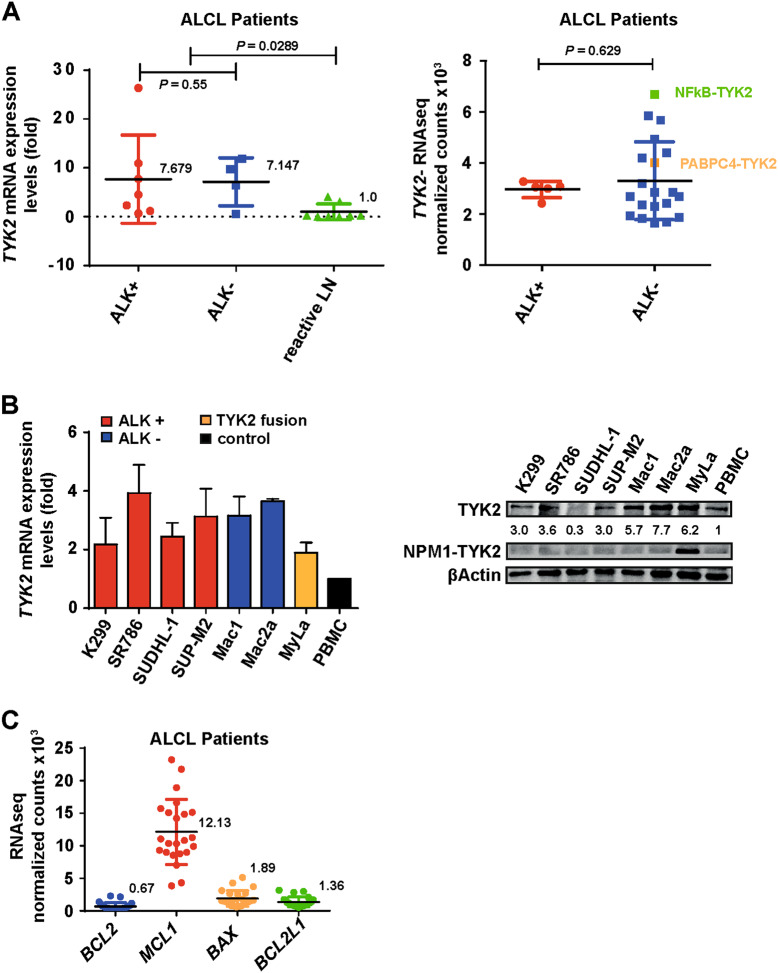
TYK2 expression is upregulated in ALCL. **a** TYK2 transcript levels were assessed by gene specific RT-PCR using RNA isolated from FFPE ALCL tumor samples. Published RNA-seq data of 23 ALCL patients were analyzed for TYK2 expression, including two cases with NFkB-TYK2 or PABC4-TYK2 fusion. **b** TYK2 expression was assessed by RT-PCR in the indicated cell lines using primers designed to recognize endogenous TYK2 only. Data are representative of the means and standard deviations of three experiments. TYK2 protein expression was analyzed in ALCL cell lines by western blot and quantified by densitometry analysis as shown by the numbers under the blot. The 81 kDa NPM1-TYK2 fusion observed in the MyLa cell line is smaller than the endogenous 134 kD TYK2 and serves as a positive control for the TYK2 antibody. **c** Published RNA-seq data of 23 ALCL patients were re-evaluated for *BCL2*, *MCL1*, *BAX*, and *BCL2L1* transcript levels, and show high levels of *MCL1* expression

TYK2 mRNA levels were analyzed using RNA isolated from formalin fixed paraffin embedded (FFPE) ALCL patient samples (7 ALCL, ALK+, 4 ALCL, ALK-, and 8 reactive lymph nodes (LN)) showing an upregulation across all ALCL samples (ALK neg: 7.1 ± 2.5, ALK pos: 7.7 ± 3.4, reactive LN 1.0 ± 0.4) as compared to lymph nodes from healthy donors (*P* = 0.0289) (Fig. 6a). These data are in line with those observed from re-evaluated, published RNA sequencing data of 23 ALCL patients (18 ALCL, ALK−, 5 ALCL, ALK+) [6]. From these data, TYK2 expression by primary ALCLs was independent of the presence or absence of ALK fusions (n.s., Fig. 6a). The highest TYK2 expression level was found in a tumor with a previously reported NFkB-TYK2 fusion protein [6]. By contrast, a PABPC4- TYK2 positive ALCL expressed TYK2 at similar levels to tumors without TYK2 fusions (Fig. 6b). Furthermore, we measured TYK2 expression in 6 ALCL cell lines (ALK+: K299, SR786, SUDHL-1, SUP-M2; and ALK−: Mac1, Mac2a), the cutaneous T-cell lymphoma cell line MyLa [31] bearing the NPM1-TYK2 fusion [5] and PBMCs. Real-time RT-PCR data revealed a 3.3-fold higher (mean 2.6 ± 0.68 SD) TYK2 expression in ALCL cell lines as compared to PBMCs (Fig. 6c). These results were recapitulated by Western blot analysis whereby TYK2 expression was 3.0- to 7.7-fold higher in ALCL cell lines as compared to PBMCs (Fig. 6b). Immunohistochemical (IHC) staining for TYK2 in ALCL patient tissue was not possible, due to the lack of specificity of commercially available antibodies for formalin-fixed tissue (Suppl. Figure 6A). However, we were able to detect enhanced TYK2 mRNA expression in FFPE sections of ALCL patients using RNA in situ hybridization (ISH) (Suppl. Figure 6B). Moreover, analysis of published RNA-seq data showed MCL1 to be expressed with a higher level of normalized counts compared to other pro-survival BCL2 family members, such as BCL2 or BCL2L1 (Fig. 6c). Hence, our data show that ALCL is a tumor that is dependent on TYK2 for cell survival, that TYK2 is activated through an autocrine loop involving IL-10 and IL-22, and that TYK2 promotes cell survival at least in part through activating the expression of the BCL2 family protein, MCL1.

## Discussion

We show here that TYK2 is expressed at high levels in human ALCL cell lines and primary ALCL patient samples. T-cell-specific loss of TYK2 in a transgenic mouse model of NPM-ALK driven lymphoma resulted in delayed tumor growth and significantly prolonged overall survival of the mice. siRNA-mediated TYK2 depletion as well as CRISPR/Cas9-mediated TYK2 disruption led to rapid induction of cell death in ALCL cells. Loss-of-TYK2 reduced pYSTAT1, pYSTAT3, and MCL1 expression. In keeping with these in vivo results in an experimental model, we found that STAT1 knockdown completely phenocopied TYK2 knockdown, whereas STAT3 knockdown only partially mirrored loss of TYK2. These results implicate a novel TYK2-STAT1 axis that is essential for tumor cell survival in ALCL. The TYK2–pYSTAT1 pathway positively regulates MCL1 expression in ALCL cells, contributing to this aberrant tumor cell survival. Knockdown of IL-10RA in ALCL cell lines resulted in growth arrest, implicating aberrantly expressed IL-10 and IL-22 in autocrine loops that provided a mechanism for aberrant TYK2 activation in tumor cells. Our results licence TYK2 as a key dependency in ALCL pathogenesis, which is potentially druggable once clinical grade TYK2 inhibitor becomes available.

The role of TYK2 in lymphoma development and progression is not yet fully understood, although, the presence of activating TYK2 fusion proteins in a small subset of ALCL patients indicates its importance [3–7]. Interestingly, re-evaluation of published RNA-seq data [6] revealed TYK2 expression in ALCL patients without TYK2 fusions at a similar level to patients bearing TYK2 fusions and at 7–8-fold higher levels than in lymph nodes from healthy donors (Fig. 6a). The mechanism responsible for enhanced expression of TYK2 in ALCLs lacking TYK2 gene fusions remains to be elucidated.

In this study, treatment with TYK2 inhibitors of ALCL cell lines combined with shRNA and CRISPR-cas9 gene disruption experiments supported STAT1 as important downstream mediator of activated TYK2 in ALCL. This is surprising, since STAT3 has widely been described as a tumor driver in ALCL [6, 32] and STAT1 has been often ascribed a tumor suppressive function by inducing cell cycle arrest, apoptosis and suppression of metastasis [33–35]. However this view was challenged recently by reports showing STAT1 was involved in radioresistance [36, 37] and as a promoter and not a suppressor in breast and gastric cancer cells [38–40]. In agreement with previous studies we find that in ALCL cell lines STAT1 is robustly expressed and phosphorylated (see Fig. 1c) [34] and loss of STAT1 has no influence on STAT3 or pSTAT3 levels despite prominent growth reduction indicating that STAT1 acts independently. Moreover, T-cell-specific TYK2 deletion in CD4-NPM- ALK transgenic mice led to reduced STAT1 phosphorylation and increased survival whereas the analogous deletion of STAT3 in the same mouse model had no effect [32]. In view of our studies, it will be important in the future to also inactivate STAT1 in T-lineage cells of this mouse ALCL model, to clearly define the dependency on STAT1.

BCL2 expression is mostly absent in ALCL, ALK+ lymphomas but MCL1 expression can be detected in the majority of these lymphomas [23, 41]. We show in this study that MCL1 expression is correlated with TYK2 expression, and TYK2 ablation in mice or human cell lines leads to markedly reduced levels of the pro-survival protein, MCL1. This is in contrast to the situation in transformed thymocytes, in which we have shown that TYK2 signals through STAT1 to upregulate BCL2. Although T-ALL and ALCL both represent transformed lymphocytes within the spectrum of the T-cell lineage, clearly signal transduction pathways are much different in developing thymocytes and memory T-cells post-antigen stimulation, which may be the cell of origin of ALCL. It is interesting that the TYK2-STAT1 signalling axis has been aberrantly rewired in each of these hematopoietic malignancies to promote cell survival, although taking advantage of different pro-survival proteins as effectors to thwart cell death at these different stages of lymphoid development.

TYK2 has previously been described in the context of IL-10, IL-12, IL-22, IL-23, and IFN type I and III signaling, as well as being linked to defective IL-12 signaling in Tyk2^−/−^ mice [29, 30, 42, 43]. Autocrine IL-10 signaling has recently been shown to upregulate TYK2 and to activate STAT1 signaling in T-ALL [3]. Among peripheral T-cell lymphomas ALCL has been associated with the highest level of IL-10 expression [44], and ALCL cells also express the IL-10 receptor, which provides an autocrine mechanism for the aberrant activation of TYK2 in ALCL cells. In this paper, we demonstrate high levels of expression of not only IL-10 but also IL-22 in ALCL cells, coincident with expression of IL10RB, IL22R1 and the common alpha chain IL10RA. Interestingly, it has been shown that the aberrant expression IL22R1 in ALCL cells are induced by NPM-ALK and mediates the pro-proliferative effect of IL-22 [45]. In ALCL patients plasma levels of IL-22 are increased, and these levels become undetectable in patients who reach complete remission [46]. Thus, both IL-10 and IL-22 are expressed by ALCL cells and form autocrine loops to activate TYK2, which along with its heterodimeric partner JAK1 provides the signaling component of both the IL-10 receptor (IL10RA/IL10RB) and the IL-22 receptor (IL10RA/IL22RA) [47].

JAK1, JAK2, and JAK3 but not TYK2 have been studied in the context of ALCL. In about 15% of ALCL, ALK- patients JAK1 (G1097) mutations and/or STAT3 (Y640) mutations have been described and lead to increased oncogenic signalling and to Ruxolitinib resistance in the latter cases [6]. JAK2 has been described to be highly phosphorylated in ALCL, ALK+ cell lines and to directly interact with NPM-ALK with potential activation of STAT5 [48]. Earlier work in ALCL cell lines has documented constitutive JAK3 phosphorylation with STAT3 activation such that apoptosis is induced when JAK3 is inhibited [49–51]. In this context it is interesting to note that TYK2, and also pan-JAK inhibitors, in our study are somewhat more effective in ALCL ALK− compared to ALK+ cell lines (Fig. 3a, b). This finding is consistent with recent work showing higher responsiveness of pSTAT3 expressing ALCL ALK- cells to JAK1-3 kinase inhibition [52].

The TYK2 inhibitors used in our study were both designed to be ATP-competitive kinase domain inhibitors, but additional inhibitor types are currently under development. Since it has been shown that TYK2 abrogation in healthy tissue attenuates but does not eliminate the effect of cytokines, it would be expected that the side effect profile of TYK2-specific inhibitors would be milder than that of pan-JAK inhibitors [53, 54]. Currently TYK2 inhibitors are being developed mostly for the treatment of autoimmune/inflammatory diseases. However, in the light of the findings in this paper and other recent reports [3, 55], it appears that TYK2 inhibitors may eventually have roles in anti-cancer treatment.

## Electronic supplementary material

Supplementary Figure 1

Supplementary Figure 2

Supplementary Figure 2

Supplementary Figure 3

Supplementary Figure 4

Supplementary Figure 5

Supplementary Figure 6

Supplementary Figure Legends

Supplementary Methods

Supplementary Table 1

Supplementary Table 2

Supplementary Table 3

Supplementary Table 4

Supplementary Table 5

Supplementary Table 6
