# Correction: Dependency on the TYK2/STAT1/MCL1 axis in anaplastic large cell lymphoma

**DOI:** 10.1038/s41375-020-0968-9

**Published:** 2020-07-14

**Authors:** Nicole Prutsch, Elisabeth Gurnhofer, Tobias Suske, Huan Chang Liang, Michaela Schlederer, Simone Roos, Lawren C. Wu, Ingrid Simonitsch-Klupp, Andrea Alvarez-Hernandez, Christoph Kornauth, Dario A. Leone, Jasmin Svinka, Robert Eferl, Tanja Limberger, Astrid Aufinger, Nitesh Shirsath, Peter Wolf, Thomas Hielscher, Christina Sternberg, Fritz Aberger, Johannes Schmoellerl, Dagmar Stoiber, Birgit Strobl, Ulrich Jäger, Philipp B. Staber, Florian Grebien, Richard Moriggl, Mathias Müller, Giorgio G. Inghirami, Takaomi Sanda, A. Thomas Look, Suzanne D. Turner, Lukas Kenner, Olaf Merkel

**Affiliations:** 1 Clinical Institute of Pathology, Department for Experimental and Laboratory Animal Pathology, Medical University of Vienna, Vienna, Austria; 2 Department of Pediatric Oncology, Dana-Farber Cancer Institute, Harvard Medical School, Boston, MA USA; 3 Unit of Laboratory Animal Pathology, University of Veterinary Medicine Vienna, Vienna, Austria; 4 Department of Oncology, Amgen Discovery Research, South San Francisco, CA 94080 USA; 5 Clinical Institute of Pathology, Medical University of Vienna, Vienna, Austria; 6 Institute of Cancer Research, Medical University of Vienna & Comprehensive Cancer Center (CCC), Vienna, Austria; 7 Department of Dermatology and Venereology, Medical University of Graz, Graz, Austria; 8 Division of Biostatistics, German Cancer Research Center (DKFZ), Heidelberg, Germany; 9 Department of Molecular Biology, Cancer Cluster Salzburg, Faculty of Natural Sciences, Paris Lodron University, Salzburg, Austria; 10 Department of Biochemistry, Christian-Albrechts-University Kiel, Kiel, Germany; 11 Ludwig Boltzmann Institute for Cancer Research (LBI-CR), Vienna, Austria; 12 Institute of Pharmacology, Center for Physiology and Pharmacology, Medical University of Vienna, Vienna, Austria; 13 Institute of Animal Breeding and Genetics, University of Veterinary Medicine Vienna, Vienna, Austria; 14 Department of Medicine I, Clinical Division of Hematology and Hemostaseology and Comprehensive Cancer Center (CCC), Medical University of Vienna, Vienna, Austria; 15 Medical University of Vienna, Vienna, Austria; 16 Pathology and Laboratory Medicine, Weill Cornell Medical College, New York, NY USA; 17 European Research Initiative for ALK related malignancies (www.erialcl.net), Vienna, Austria; 18 Cancer Science Institute of Singapore, National University of Singapore, 117599 Singapore, Singapore; 19 Division of Cellular and Molecular Pathology, Department of Pathology, University of Cambridge, Cambridge, UK; 20 CBMed Core Lab2, Medical University of Vienna, Vienna, Austria

**Keywords:** Targeted therapies, T-cell lymphoma

Correction to: *Leukemia*

10.1038/s41375-018-0239-1

This article was originally published under NPG’s License to Publish, but has now been made available under a CCBY 4.0 license. Furthermore, since publication of the original paper the authors have agreed to add Christina Sternberg as an author. The PDF and HTML versions of the paper have been modified accordingly.

