# Supplementary figures and images for: Dependency on the TYK2/STAT1/MCL1 axis in anaplastic large cell lymphoma
Source: Leukemia. 2018 Aug 21;33(3):696–709. doi: 10.1038/s41375-018-0239-1 (PMC8076043; doi:10.1038/s41375-018-0239-1)

# Supplementary Figure 1

**A**

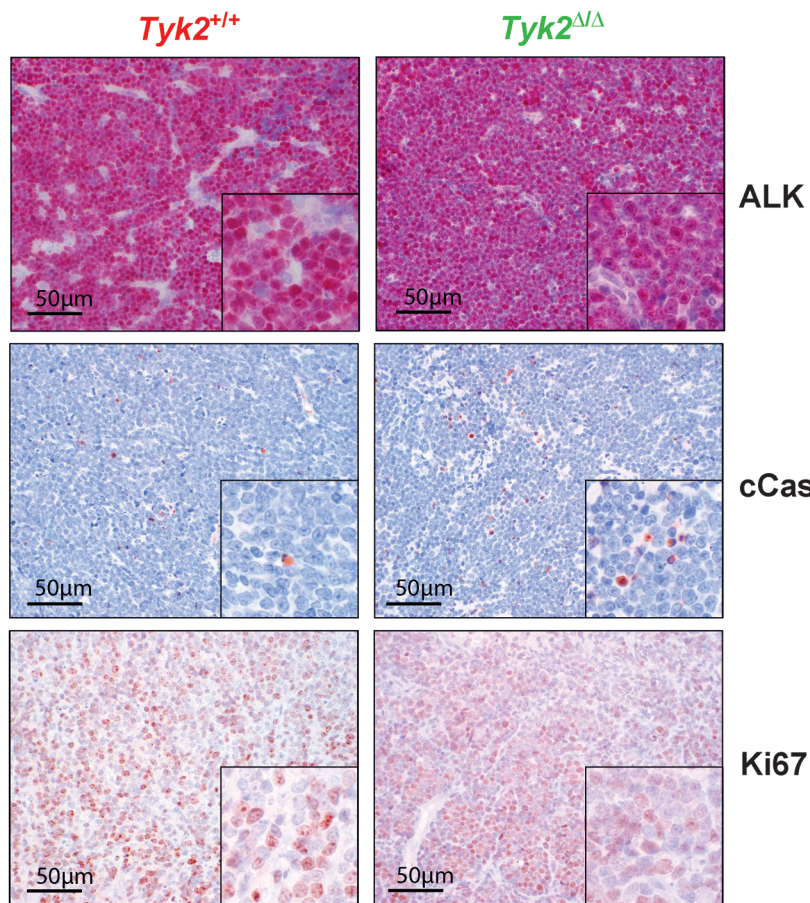

**B**

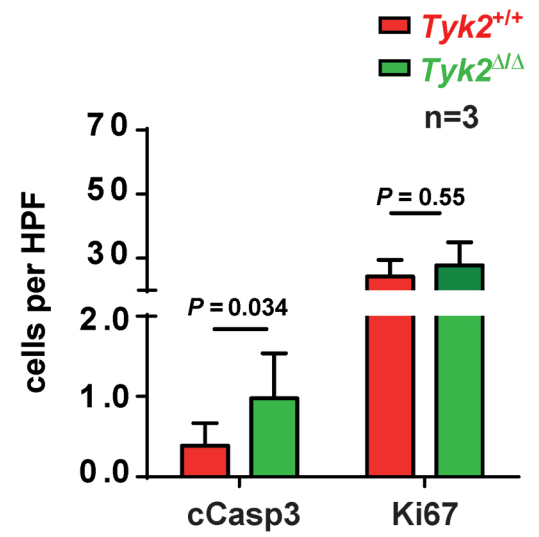

**C**

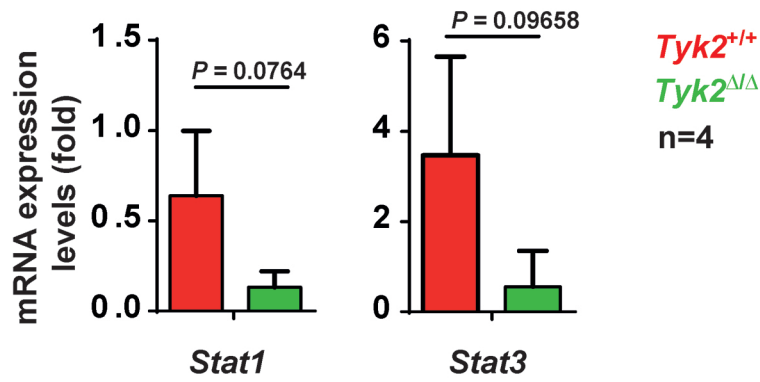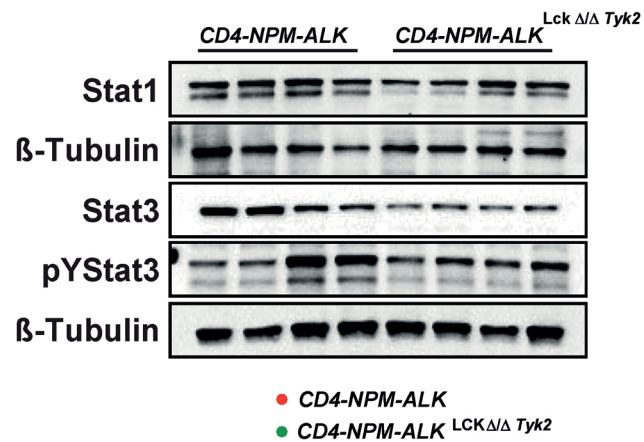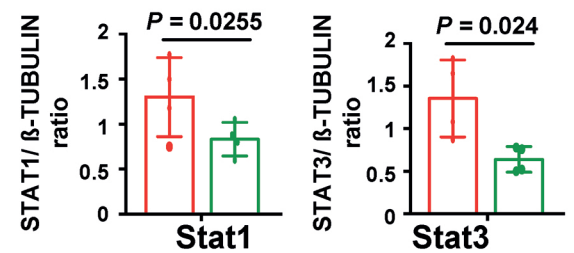

**D**

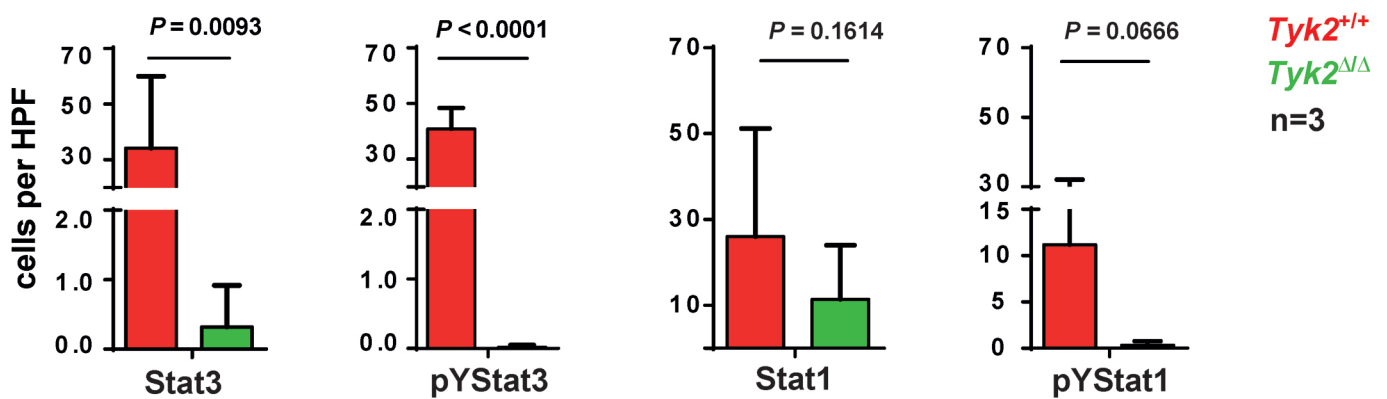

Supplement: Supplementary file 1 — Supplementary Figure 1 [file 41375_2018_239_MOESM1_ESM.pdf]

# Supplementary Figure 2

**A**

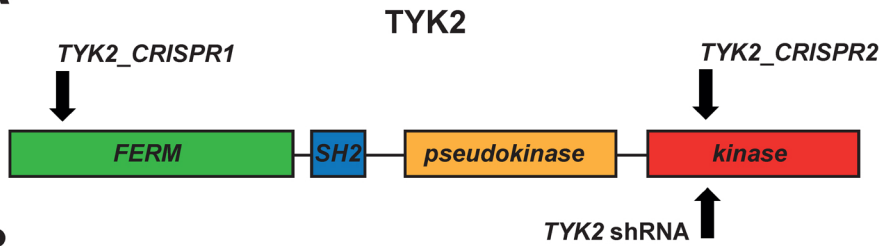

**B**

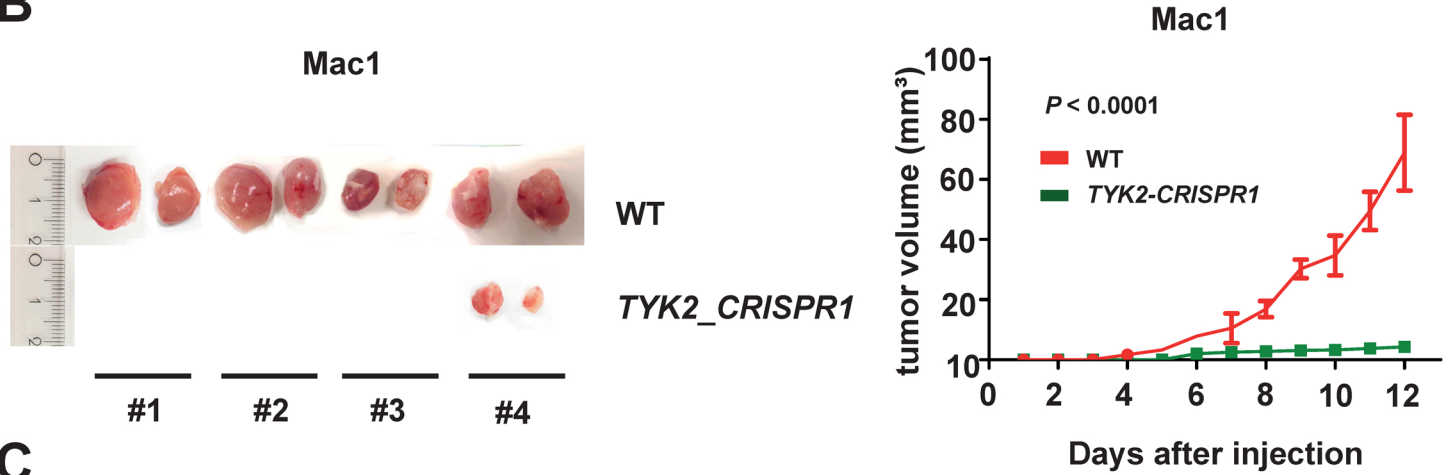

**C**

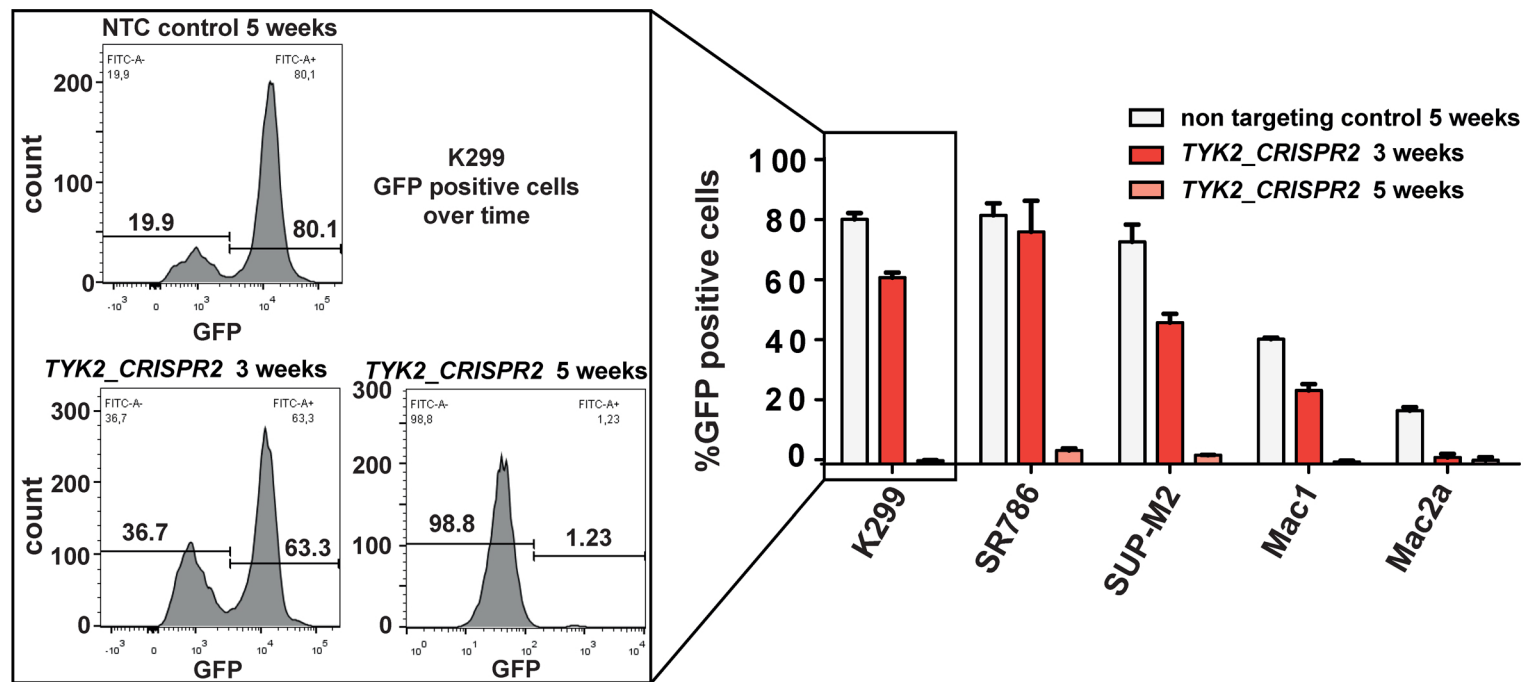

**D**

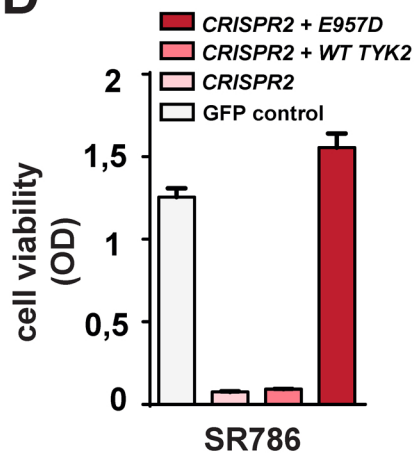

Supplement: Supplementary file 2 — Supplementary Figure 2 [file 41375_2018_239_MOESM2_ESM.pdf]

# Supplementary Figure 2

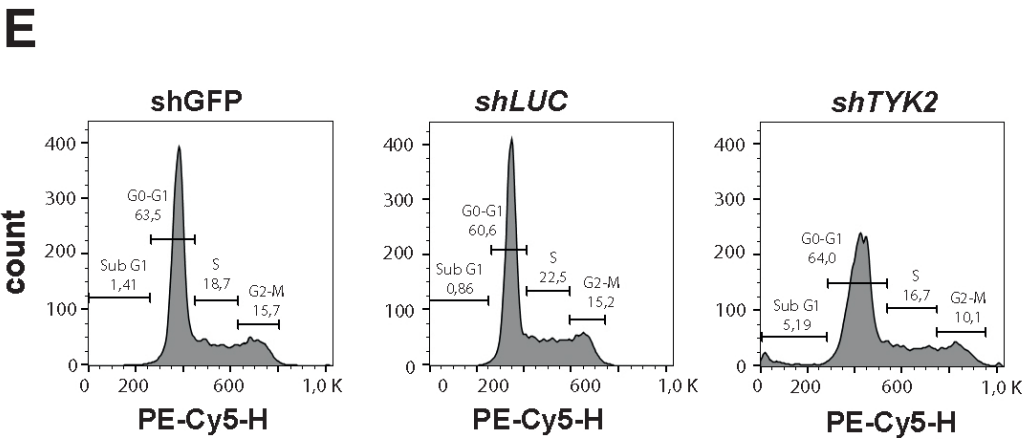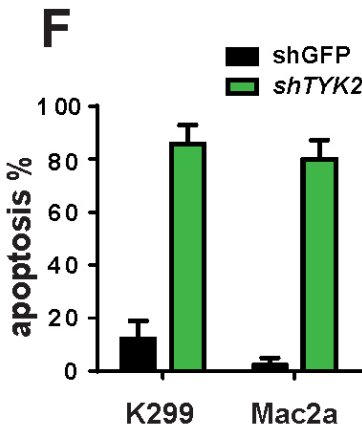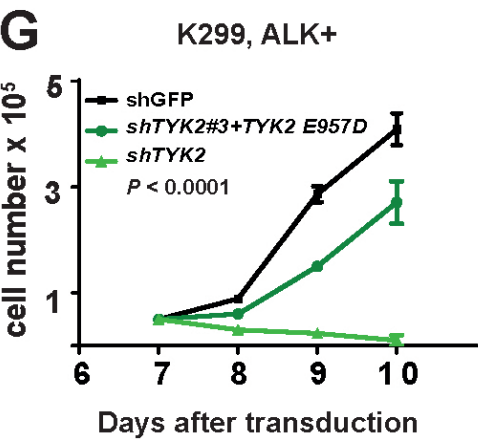

Supplement: Supplementary file 3 — Supplementary Figure 2 [file 41375_2018_239_MOESM3_ESM.pdf]

# Supplementary Figure 3

A

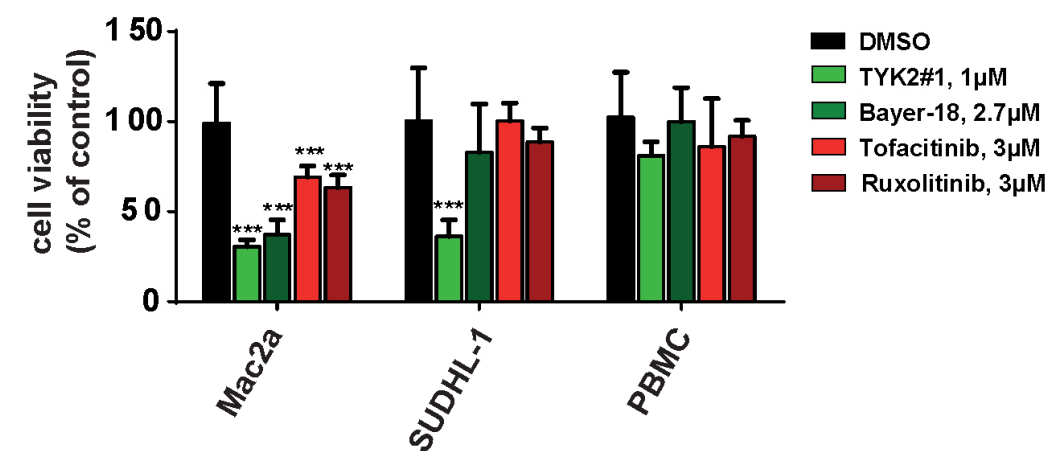

B

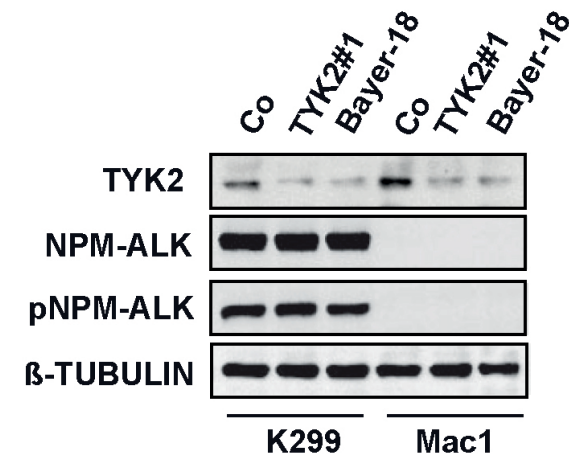

C

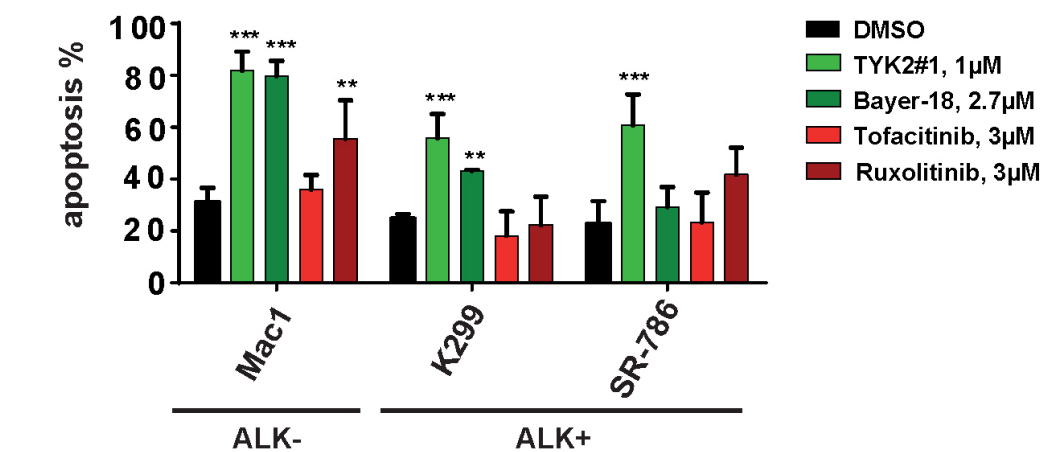

Supplement: Supplementary file 4 — Supplementary Figure 3 [file 41375_2018_239_MOESM4_ESM.pdf]

# Supplementary Figure 4

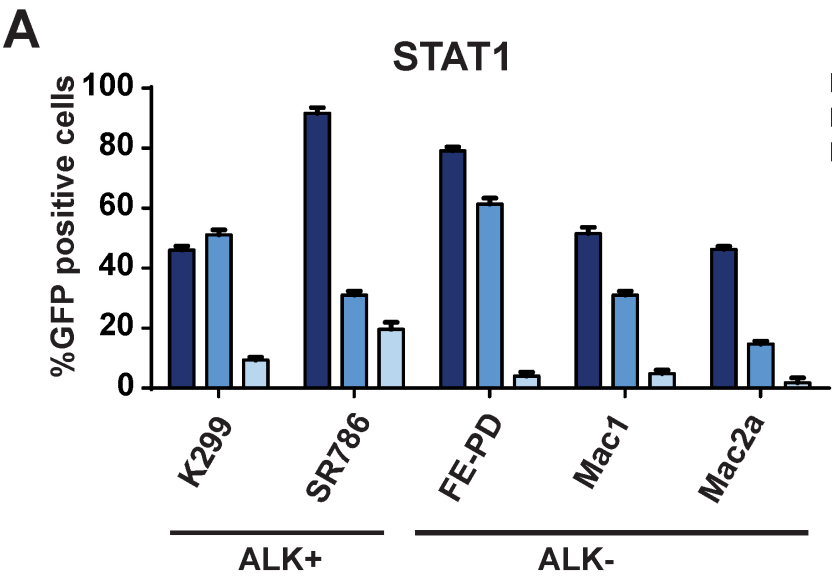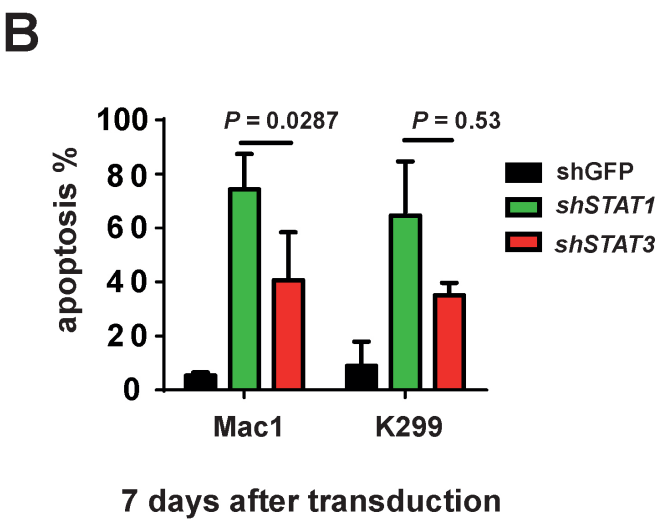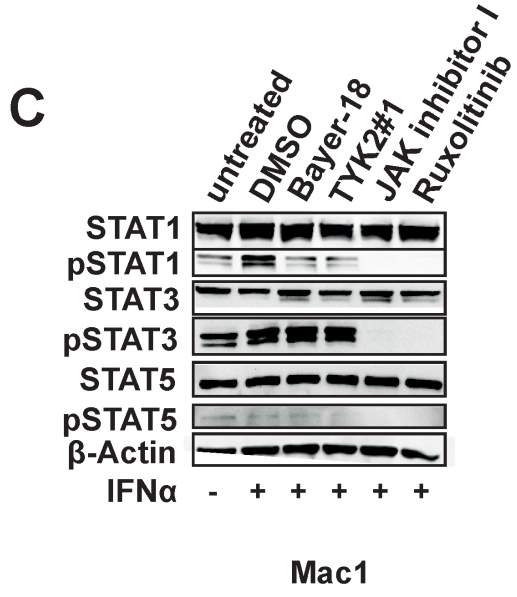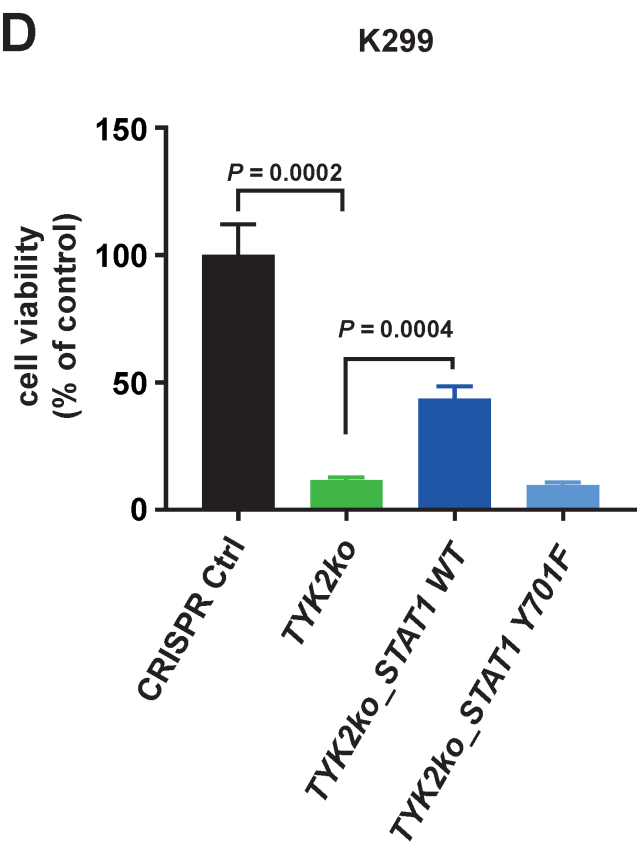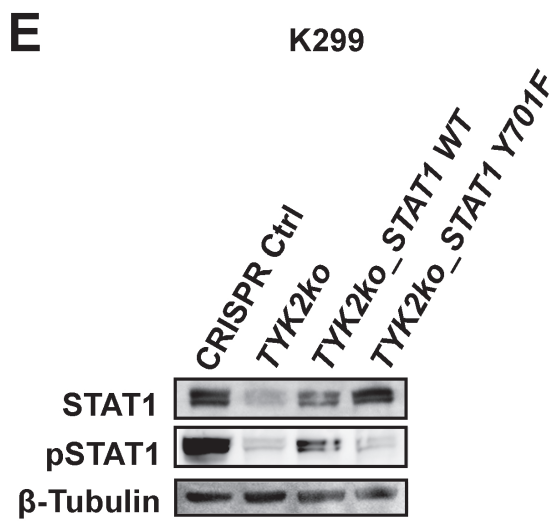

Supplement: Supplementary file 5 — Supplementary Figure 4 [file 41375_2018_239_MOESM5_ESM.pdf]

# Supplementary Figure 5

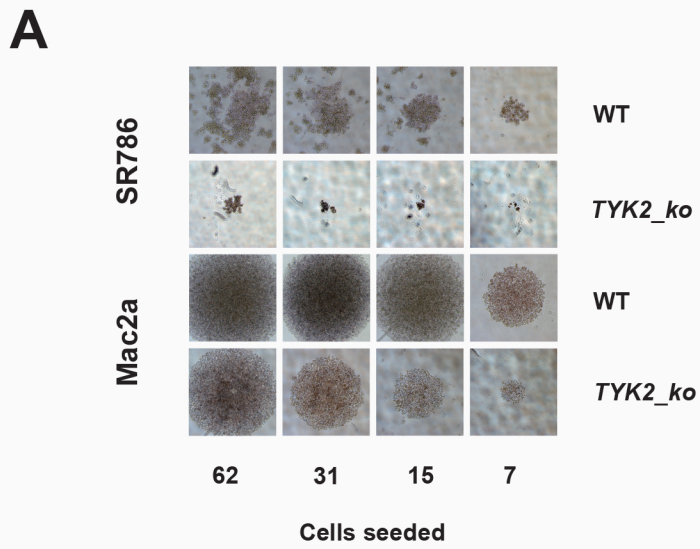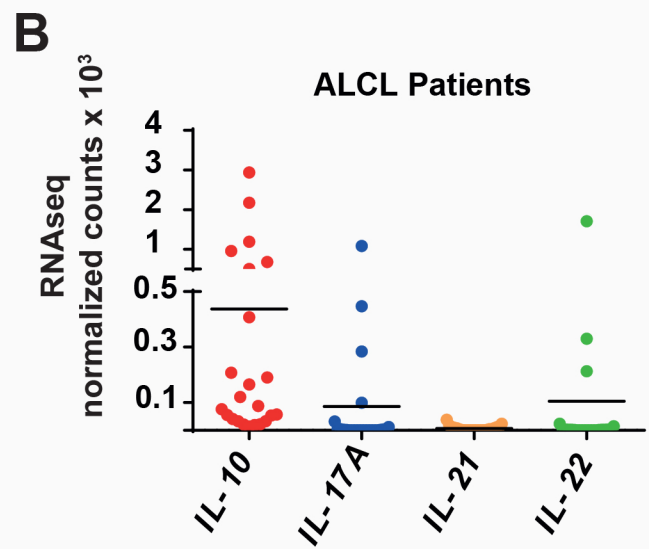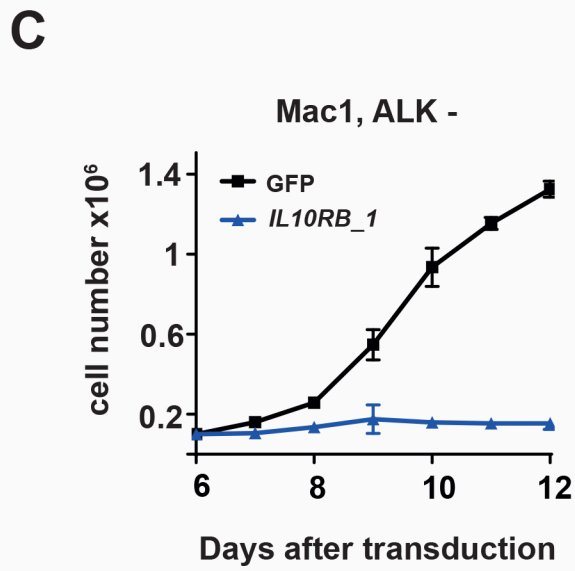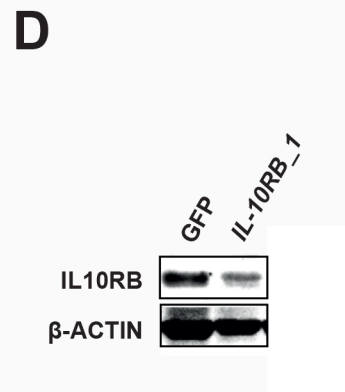

Supplement: Supplementary file 6 — Supplementary Figure 5 [file 41375_2018_239_MOESM6_ESM.pdf]

# Supplementary Figure 6

A

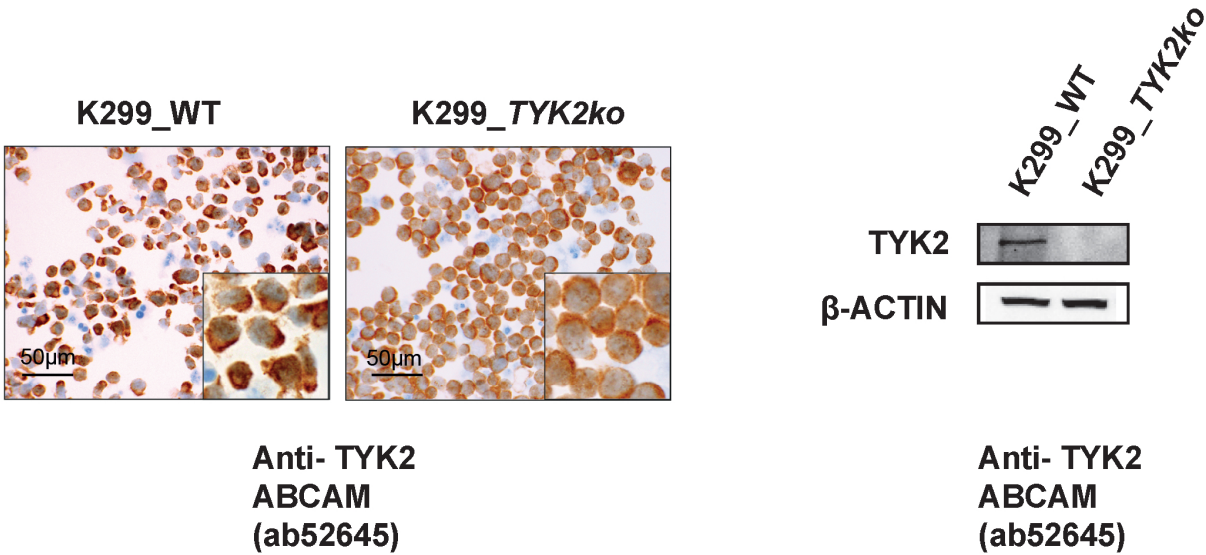

B

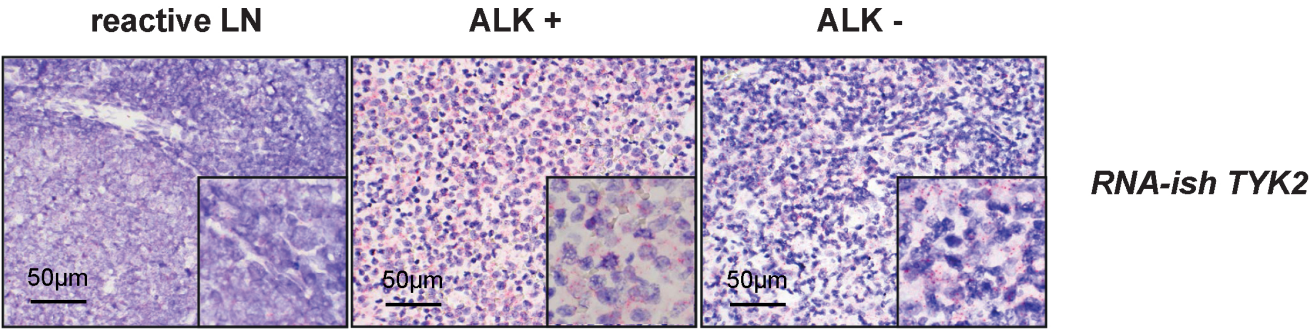

*RNA-ish TYK2*

Supplement: Supplementary file 7 — Supplementary Figure 6 [file 41375_2018_239_MOESM7_ESM.pdf]
