# Supplementary material for: Dependency on the TYK2/STAT1/MCL1 axis in anaplastic large cell lymphoma
Source: Leukemia. 2018 Aug 21;33(3):696–709. doi: 10.1038/s41375-018-0239-1 (PMC8076043; doi:10.1038/s41375-018-0239-1)
Supplement: Supplementary file 8 — Supplementary Figure Legends [file 41375_2018_239_MOESM8_ESM.pdf]

Figure S1: Alk, Stats, cleaved Caspase 3 and Ki67 expression as well as cytokine production in lymphoma tissues from *CD4*-NPM-ALK and *CD4*-NPM-ALK$^{LCK\Delta\Delta Tyk2}$ mice (A) IHC of lymphoma tissues from *CD4*-NPM-ALK and *CD4*-NPM-ALK$^{LCK\Delta\Delta Tyk2}$ mice using antibodies against Alk, cleaved Caspase 3 (CC3) or Ki67. (B) Quantification of CC3 or Ki67 in sections of *CD4*-NPM-ALK and *CD4*-NPM-ALK$^{LCK\Delta\Delta Tyk2}$ lymphomas. The mean numbers of positive cells per high-power field (HPF) among 2,000 cells per tumor sample were determined using TissueQuest software. Data are mean values ± s.d. of three mice. (C) mRNA and protein expression of *Stat1* and *Stat3 in CD4*-NPM-ALK or *CD4*-NPM-ALK$^{LCK\Delta\Delta TYK2}$ lymphomas. Data are mean values ± s.d. of four mice. (D) Assessment of Stat3, pYStat3, Stat1 and pYStat1 in sections of *CD4*-NPM-ALK and *CD4*-NPM-ALK$^{LCK\Delta\Delta Tyk2}$ lymphomas. The mean numbers of positive cells HPF among 2,000 cells per tumor sample were determined using TissueQuest software. Data are mean values ± s.d. of three mice.

Figure S2: Effects of TYK2 deletion *in vivo* and *in vitro* and rescue experiments. (A) Domain structure of TYK2 indicating the respective sgRNA (CRISPR) and shRNA target sites. Exact sequence of guideRNAs and shRNAs can be found in Table S2,3 (B) TYK2 CRISPR knockout reduces cell growth *in vivo*. Mac1 cells with or without TYK2 expression were injected subcutaneously into the hind flanks of NSG mice; the mice were sacrificed after 14 days, tumors excised and photographed. Tumor size *in vivo* was determined daily with callipers until day 12 after injection. Data represent the means of four mice. (C) The indicated cell lines were transduced with GFP labeled sgRNA targeting TYK2 in the kinase domain (TYK2_CRISPR2) or a non-targeting control sgRNA (NTC). GFP expression was measured by flow cytometry 3 and 5 weeks after transduction. (D) Viability of the ALK positive cell line SR786 with (CRISPR2) and without TYK2 deletion transduced with the indicated constructs and assessed by XTT assay. (E) On day 7 after transduction with respective shRNAs (shGFP, shLUC or shTYK2) cells either underwent methanol fixation and were stained with PI for cell cycle analysis or (F) were stained with Annexin V-FITC to monitor apoptosis and analyzed by flow cytometry. Means +/- SEM of two experiments are shown. (G) The ALK positive cell line Karpas-299 was transduced either with a non-targeting plasmid (NTC) or with a TYK2 plasmid carrying the E957D mutation in the kinase domain before TYK2 ablation by shRNA (shTYK2).

Figure S3: TYK2 or panJAK inhibition in ALCL cells (A) The indicated cell lines were treated with the TYK2 inhibitors TYK2#1 (1µM) or Bayer-18 (2.7 µM), or the pan-JAK inhibitors Ruxolitinib

(3 µM) or Tofacitinib (3 µM) for 72 hours. DMSO was used as a control. Cell proliferation was assessed by an XTT assay. (B) The indicated cell lines were treated with the TYK2 inhibitors TYK2#1 (1µM) or Bayer-18 (2.7µM) for 48 hours. Whole cell extracts were subjected to immunoblot analyses with antibodies against TYK2, NPM-ALK, pNPM-ALK and β-Tubulin. (C) Apoptotic cell death was monitored by AnnexinV-FITC staining 72 hours after treatment with the indicated inhibitors. Means +/- SEM of two experiments are shown. P-values by the Student's t-test.

Figure S4: ALCL cells depend on STAT1 for survival. (A) The indicated cell lines were transduced with GFP labeled sgRNA targeting STAT1 (CRISPR_STAT1) or a non-targeting control sgRNA into Cas9 expressing ALCL cell lines. GFP expression was measured by flow cytometry 3 and 5 weeks after transduction. (B) Apoptosis was measured 7 days following transduction with the indicated shRNA by flow cytometric analysis of cells stained with Annexin V-FITC. Means +/- SEM of two experiments are shown. (C) The ALK negative cell line Mac1 was treated with TYK2 inhibitors and pan-JAK inhibitors for 3 hours before incubation with 100 U/ml INF-α for 10 minutes. Whole cell extracts were subjected to immunoblot analyses with antibodies against STAT1, pYSTAT1, STAT3, pYSTAT3, STAT5, pYSTAT5 and β-ACTIN. (D) The ALK positive cell line Karpas-299 that was transduced with sgRNA targeting *TYK2* (K299 TYK2ko) , was additionally transduced with either WT STAT1 plasmid (STAT1 WT) or with a STAT1 plasmid carrying the Y701F mutation leading to an un-phosphorylated STAT1 (STAT1 Y701F). Cell viability was compared between the indicated cell lines using cell titer glow 1 week after viral transduction. (E) Whole cell extracts were subjected to immunoblot analyses and stained with antibodies against STAT1, pYSTAT1 and β-Tubulin.

Figure S5: Expression of cytokines and IL10RB knock-down in ALCL cells. (A) Limiting dilution of the ALK-positive cell line SR786 or the ALK-negative cell line Mac2a with and without CRISPR-Cas9 *TYK2* knockout in 96-well plates containing RPMI1640 and 10% FCS. *TYK2* knockout (TYK2_ko) cells require greater plating cell numbers for cell growth assessed after 2 weeks of incubation. (B) IL-10 (red), IL-17A (blue), IL-21 (yellow) and IL-22 (green) expression in 25 ALCL patient samples was determined using published RNA-Seq. data. Relative abundance is expressed as normalized counts (16). (C) Knockdown of IL10RB in the ALK negative cell line Mac1. Cells were counted 6, 7, 8, 9, 10 and 12 days after transduction. (D) Cells were subjected

to immunoblot analyses with antibodies against IL-10RB and β-ACTIN.

Figure S6: TYK2 expression in ALCL patient samples. (A) IHC staining of cytospins from the ALK positive cell line Karpas-299 with and without TYK2 knockout with the anti-TYK2 antibody ab52645 (Abcam). Stainings show no difference between WT and TYK2 knockout due to the lack of TYK2 specificity in IHC of commercially available antibodies (one representative example shown). TYK2 knockout in Karpas-299 cells has been confirmed by Western blot as well as Sanger sequencing. (B) RNAish for TYK2 (red) transcripts in FFPE ALCL sections and a reactive lymph node, representative samples are shown of triplicates analyzed.
