# Supplementary material for: Dependency on the TYK2/STAT1/MCL1 axis in anaplastic large cell lymphoma
Source: Leukemia. 2018 Aug 21;33(3):696–709. doi: 10.1038/s41375-018-0239-1 (PMC8076043; doi:10.1038/s41375-018-0239-1)
Supplement: Supplementary file 9 — Supplementary Methods [file 41375_2018_239_MOESM9_ESM.pdf]

Supplementary Methods

Quantitative RT-PCR

Total RNA was extracted using RNeasy FFPE kit (Quiagen, Hilden, Germany) for FFPE quantification or RNeasy kit (Quiagen) for ALCL cell lines or for murine tumor samples. Specific primers for gene detection are shown in Table S1A and S1B. RNA samples were transcribed using a Reverse Aid First Strand cDNA Synthesis Kit (Thermo Scientific, Waltham, MA). For Quantitative PCR analysis using cyber green the AB 7300 system was used (Thermo Scientific).

Murine lymphoma models

Animal experiments were carried out according to an ethical animal license protocol that was approved by the Medical University of Vienna and Austrian Ministry authorities (BMWFW-66.009/0389-WF/V/3b/2014). NSG mice were purchased from the National Cancer Institute (NCI; Frederick, MD) (*NOD/SCID/IL-2RγKO* (NSG) and $8x10^5$ cells suspended in 100 µl of PBS, 0.2% BSA were injected sub-cutaneously (s.c.) into the hind flanks. Mice carrying the human NPM-ALK cDNA expressed under the *CD4* promoter [1, 2] (mixed background) were crossed with mice that were intercrossed from *LCK*-Cre mice (C57BL/6) and mice carrying *loxP*-flanked *TYK2 (*C57BL/6*)* [3]. Mice were genotyped using primers listed in Table S1D to distinguish between ALK positive and ALK negative (ALK), LCK-Cre positive and negative (LCK) and TYK2 WT or floxed (TYK2 loxp) mice. For the Kaplan-Meier curve, only isogenic littermates of the following genotypes were used: wild-type control (WT), *CD4-NPM-ALK-TYK2*$^{flox/flox}$ (*CD4*-NPM-ALK), *CD4-NPM-ALK-CD4-TYK2* $^{\Delta/\Delta}$ (*CD4*-NPM-ALK$^{LCK\Delta\Delta TYK2}$) mice. Mice were sacrificed upon signs of tumor development (which usually led to death within 1-2 days). Tumors were excised and also organs including spleen, liver, kidney and lungs. These were formalin-fixed and paraffin-embedded or frozen in liquid nitrogen for further analysis.

shRNA sources

pLKO.1-puro vectors containing selected shRNAs targeting TYK2, STAT1, IL-10R1, IL-10R2 and plasmids expressing TYK2-E957D or TYK2-WT were kindly provided by Takaomi Sanda (CSI, Singapore). shRNA sequences are listed in Table S2. Viral supernatants containing shRNA constructs targeting STAT3 or non-targeting scrambled shRNA were kindly provided by Fritz Aberger, Paris Lodron University, Salzburg, Austria. Viruses were generated and ALCL cell lines infected as previously described [2, 4].

CRISPR/Cas9 genome editing

TYK2 deletion in the FERM domain (TYK2-CRISPR1), kinase-domain (TYK2-CRISPR2) or STAT1 (STAT1-CRISPR) was achieved by using CRISPR/Cas9 genome editing technology. Guide RNAs were designed by using the CRISPR design tool (http://crispr.mit.edu) and are listed in Supplementary Table 3. For TYK2-CRISPR1, the guide RNA was annealed and cloned into the BbsI sites of the lentiCRISPR v2 vector (Addgene # 52961). Then virus was produced and ALCL cells were transduced as described before [2]. After two weeks, limiting dilution in conditioned media was performed to obtain single cell clones. Genomic DNA from single clones was isolated using the DNeasy kit (Quiagen) and respective regions in TYK2 amplified and deletions confirmed by Sanger sequencing (see Table S1C for primer sequences) and Western blot. To generate TYK2-CRISPR2 knockouts, cells were first transduced with lentiCas9Blast (Addgene #52962) using Blasticidin selection to express Cas9. Then we cloned a GFP marker cassette into the lentiGuide-Puro vector (Addgene #52963) containing either guide RNAs targeting TYK2 or STAT1 (Table S3: TYK2-CRISPR2, STAT1-CRISPR). Then virus was produced and ALCL cells were transduced as previously described [2].

pMSCV-NP-hSTAT1-Y701F-FLAG and MIG-NP-hSTAT1-wt-FLAG were kindly provided by Takaomi Sanda [2]. hSTAT1-Y701F and hSTAT1-wt retroviral vectors were co-transfected into 293 T cells with packaging plasmid pMD-MLV and envelope plasmid VSV-G. After 48 h, viral supernatants were collected and passed through a 0.45 μm filter (Millipore). Target cells were re-suspended in 1ml of viral supernatant in the presence of Polybrene (8 μg/ml). The plates were centrifuged (2000 g, 1.5 h) and then set in an incubator. After 3 days, the cells were expanded and isolated by FACS.

Flow cytometry

Transfection efficiency and number of GFP positive cells was determined using FACSCanto (BD Biosciences, San Jose, CA, USA) using FACSDiva software version 8.0. Data were analyzed with FlowJo Vx.07. Apoptosis was detected with Annexin V coupled to FITC. Cell cycle analysis was performed according to standard protocols using Propidium Iodide to stain methanol fixed cells.

Immunohistochemistry

For immunohistochemical analysis, tissue was prepared and proceeded as described before [5]. Sections were stained using antibodies listed in Supplementary table 5. 3 ALK+ and 3 ALK- ALCL FFPE tissue specimens together with 2 reactive lymphnode samples as control were externally stained with RNA-ISH technology by Sophistolab, Switzerland (http://www.sophistolab.ch/site/site.asp).

Cytokine arrays

A ProcartaPlex™ Multiplex immunoassay (Affymetrix eBioscience, San Diego, CA) detecting IFN-α, IFN-γ, IL-9, IL-10, IL-17A, IL-21, IL-22, IL-23, IL-27 was performed according to manufacturer's instructions on the supernatants of ALCL cell lines Karpas-299 and Mac1 with and without TYK2 knockout. Samples were measured using the Bio-Plex 200 multiplex suspension system in combination with Bio-Plex Manager software, version 6.1, using five parametric curve fitting (Bio-Rad, Hercules, CA).

1. Chiarle R, Gong JZ, Guasparri I, Pesci A, Cai J, Liu J*, et al.* NPM-ALK transgenic mice spontaneously develop T-cell lymphomas and plasma cell tumors. *Blood* 2003 3/1/2003; **101**(5)**:** 1919-1927.

2. Sanda T, Tyner JW, Gutierrez A, Ngo VN, Glover J, Chang BH*, et al.* TYK2-STAT1-BCL2 pathway dependence in T-cell acute lymphoblastic leukemia. *Cancer discovery* 2013 May; **3**(5)**:** 564-577.

3. Vielnascher RM, Hainzl E, Leitner NR, Rammerstorfer M, Popp D, Witalisz A*, et al.* Conditional ablation of TYK2 in immunity to viral infection and tumor surveillance. *Transgenic Res* 2014 Jun; **23**(3)**:** 519-529.

4. Kasper M, Regl G, Eichberger T, Frischauf AM, Aberger F. Efficient manipulation of Hedgehog/GLI signaling using retroviral expression systems. *Methods Mol Biol* 2007; **397:** 67-78.

5. Laimer D, Dolznig H, Kollmann K, Vesely PW, Schlederer M, Merkel O*, et al.* PDGFR blockade is a rational and effective therapy for NPM-ALK-driven lymphomas. *Nature medicine* 2012 Nov; **18**(11)**:** 1699-1704.
