# Supplementary material for: Dependency on the TYK2/STAT1/MCL1 axis in anaplastic large cell lymphoma
Source: Leukemia. 2018 Aug 21;33(3):696–709. doi: 10.1038/s41375-018-0239-1 (PMC8076043; doi:10.1038/s41375-018-0239-1)
Supplement: Supplementary file 10 — Supplementary Table 1 [file 41375_2018_239_MOESM10_ESM.pdf]

Table S1

A: Human RT-PCR primer sequences
(gene specific RT primers for FFPE tissue)

Target Gene	Sequence	ID
TYK2_1	CGGGTTGACCAGAAGAT	Hu endogenous TYK2
TYK2_2	CGCAGGCGGCCCTCATACA	Hu endogenous TYK2
TYK2_3	GGACCCTGAGGAGGGC	gene specific TYK2_RT
GAPDH_1	TCTCCTCTGACTTCAACA	huGAPDH fw
GAPDH_2	ACCACCCTGTTGCTGTAG	huGAPDH rev
GAPDH_3	GTCATACCAGGAAATGAG	gene specific GAPDH_RT

B: Murine RT-PCR primer sequences.

Target Gene	Sequence	ID
TYK2	CCCTTGGATGTGTGTTACGG	TYK2 mouse Fw
TYK2	TGTTCCGGCCACATACATTCCC	TYK2 mouse Rev
MCL1	ACTGTTGGCGTGTTATGCTC	MCL1 mouse Fw
MCL1	AGGAAAGCTGTGCTGACTCT	MCL1 mouse Rev
STAT1	AAATCTTTGGGCATTTTCCA	STAT1mouseFw
STAT1	GTACAGCCGCTTTTCTCTGG	STAT1mouseRev
STAT3	AATGTCCTCTATCAGCACAACC	STAT3mouseFw
STAT3	TCTCCACCACCTTCATTTTCTG	STAT3mouseRev
BCL-2	GAGCGTCAACAGGGAGATGT	BCL2mouseFw
BCL-2	CATGCTGGGGCCATATAGTT	BCL2mouseRev
GAPDH	GTTGTCTCCTGCGACTTCA	GAPDH mouse Fw
GAPDH	GGTGGTCCAGGGTTTCTTA	GAPDH mouse Rev

C: TYK2 CRISPR1 PCR primer sequences.

Target Gene	Sequence	ID
TYK2_4	CTGCGCCTTCGGAACGTC	huCRISPR1fw
TYK2_5	CCAGAGCCATGTGGGGAG	huCRISPR1rev

D: Genotyping PCR primer sequences for murine samples.

Target Gene	Sequence	ID
ALK	TCCCTTGGGGGCTTTGAAATAACACC	ALKfw
ALK	CGAGGTGCGGAGCTTGCTCAGC	ALKrev
LckCRE	CCTCCTGTGAACTTGGTGCTTGAG	LckCreF
LckCRE	TGCATCGACCGGTAATGCAG	LckCreR
Myogen	TTACGTCCATCGTGGACAGC	myogF
Myogen	TGGGCTGGGTGTTAGCCTTA	myogrk
TYK2 loxp	GCAAGCCTGGGTTACATGAG	FLK5 new
TYK2 loxp	TGGACTGGAACTTGTGAGGA	FLK3 new
