# Supplementary material for: Dependency on the TYK2/STAT1/MCL1 axis in anaplastic large cell lymphoma
Source: Leukemia. 2018 Aug 21;33(3):696–709. doi: 10.1038/s41375-018-0239-1 (PMC8076043; doi:10.1038/s41375-018-0239-1)
Supplement: Supplementary file 11 — Supplementary Table 2 [file 41375_2018_239_MOESM11_ESM.pdf]

Table S2

Targeting sequences for shRNAs.

Target Gene	Clone ID	Clone Name	Target Sequence	ID
JAK1	TRCN0000121212	NM_002227.1-3102s1c1	CTTCGGTTTAACCAAAGCAAT	JAK1-shRNA #5
JAK1	TRCN0000121213	NM_002227.1-693s1c1	GCGATATATTCCAGAAACATT	JAK1-shRNA #6
TYK2	TRCN0000003124	NM_003331.x-3209s1c1	CGAGCACATCATCAAGTACAA	TYK2-shRNA #3
STAT1	TRCN0000004264	NM_007315.x-1201s1c1	GAACAGAAATACACCTACGAA	STAT1-shRNA #1
STAT1	TRCN0000004265	NM_007315.x-2339s1c1	CCCTGAAGTATCTGTATCCAA	STAT1-shRNA #2
IL10RA	TRCN0000058731	NM_001558.2-1275s1c1	AGTGGCATTGACTTAGTTCAA	IL10RA-shRNA #4
IL10RA	TRCN0000058732	NM_001558.2-462s1c1	GTGAACCTAGAGATCCACAAT	IL10RA-shRNA #5
IL10RB	TRCN0000058266	NM_000628.3-945s1c1	CTTTCCATTGTCGGATGAGAA	IL10RB-shRNA#4
IL10RB	TRCN0000058267	NM_000628.3-387s1c1	GCATTCAGACTGGGTAAACAT	IL10RB-shRNA#5
GFP	-	-	ACAACAGCCACAACGTCTATA	GFP shRNA
Luc	-	-	CTTCGAAATGTCCGTTCGGTT	Luc shRNA
