# Supplementary material for: Dependency on the TYK2/STAT1/MCL1 axis in anaplastic large cell lymphoma
Source: Leukemia. 2018 Aug 21;33(3):696–709. doi: 10.1038/s41375-018-0239-1 (PMC8076043; doi:10.1038/s41375-018-0239-1)
Supplement: Supplementary file 12 — Supplementary Table 3 [file 41375_2018_239_MOESM12_ESM.pdf]

Table S3

GuideRNA Sequences.

Target Gene	Sequence	ID
TYK2	GGGGTGGCCCCTACAGACCC	TYK2_CRISPR1
TYK2	CTGGTCAAGATCGGGGACTT	TYK2_CRISPR2
STAT1	GAATGAGGGTCCTTTGGGAA	STAT1_CRISPR
GFP	CACCGGTGAACCGCATCGAGCTGAA	GFP control (CRISPR1)
NTG	GGATGATAACTGGTCCGCAGTGG	non targeting control (CRISPR2)
