# Supplementary material for: Dependency on the TYK2/STAT1/MCL1 axis in anaplastic large cell lymphoma
Source: Leukemia. 2018 Aug 21;33(3):696–709. doi: 10.1038/s41375-018-0239-1 (PMC8076043; doi:10.1038/s41375-018-0239-1)
Supplement: Supplementary file 13 — Supplementary Table 4 [file 41375_2018_239_MOESM13_ESM.pdf]

Table S4

Antibodies for Immunoblotting.

Target Protein	ID	Company	Dilution
TYK2	#3912S	Cell Signaling, Danvers, MA, US	1:100
JAK1	#3344S	Cell Signaling, Danvers, MA, US	1:100
STAT1	#9172S	Cell Signaling, Danvers, MA, US	1:100
phospho-STAT1	#Y701	Cell Signaling, Danvers, MA, US	1:100
STAT3	#D3Z2G	Cell Signaling, Danvers, MA, US	1:100
phospho-STAT3	#9131	Cell Signaling, Danvers, MA, US	1:100
STAT5	#9352	Cell Signaling, Danvers, MA, US	1:100
Phispho-STAT5	#C11C5	Cell Signaling, Danvers, MA, US	1:100
ALK	#3633	Cell Signaling, Danvers, MA, US	1:100
beta-Actin	#4967	Cell Signaling, Danvers, MA, US	1:100
MCL1	#SC-819	Santa Cruz, Dallas, Texas	1:100
IL-10RA	#ab94811	Abcam, Cambridge, UK	1:100
IL-10RB	#ab106282	Abcam, Cambridge, UK	1:100
HRP-conjugated secondary mouse	315-0035-008	Westgrove, PA, USA	1:10000
HRP-conjugated secondary rabbit	111-036-047	Westgrove, PA, USA	1:10000
