# Supplementary material for: Dependency on the TYK2/STAT1/MCL1 axis in anaplastic large cell lymphoma
Source: Leukemia. 2018 Aug 21;33(3):696–709. doi: 10.1038/s41375-018-0239-1 (PMC8076043; doi:10.1038/s41375-018-0239-1)
Supplement: Supplementary file 14 — Supplementary Table 5 [file 41375_2018_239_MOESM14_ESM.pdf]

Table S5

Antibodies for Immunohistochemistry.

Target Protein	ID	Company	Dilution
Ki67	NCL-KI67-P	Novocastra, Leica Biosystems, Wetzlar, Germany	1:1000
cleaved caspase 3	#9661	Cell Signaling, Danvers, MA, US	1:100
STAT3	sc-7179	Santa Cruz, Dallas, Texas	1:200
phospho-STAT3	#9145	Cell Signaling, Danvers, MA, US	1:80
STAT1	#9175	Cell Signaling, Danvers, MA, US	1:100
phospho-STAT1	#9167	Cell Signaling, Danvers, MA, US	1:100
ALK	51-3900	Cell Signaling, Danvers, MA, US	1:50
