# Supplementary material for: Dependency on the TYK2/STAT1/MCL1 axis in anaplastic large cell lymphoma
Source: Leukemia. 2018 Aug 21;33(3):696–709. doi: 10.1038/s41375-018-0239-1 (PMC8076043; doi:10.1038/s41375-018-0239-1)
Supplement: Supplementary file 15 — Supplementary Table 6 [file 41375_2018_239_MOESM15_ESM.pdf]

Table S6

TYK2/JAK inhibitors.

Target Protein	ID	Company	Concentration
TYK2	Bayer-18	Symansis, New Zealand	2.7 μM
TYK2	TYK2#1	Genentech, San Francisco, CA, USA	1 μM
JAK1/JAK2	Ruxolitinib	Santa Cruz Biotechnology, Dallas, Texas, USA	3μM
JAK1/JAK3	Tofacitinib	Selleckchem, Houston, Texas, USA	3μM
